# Complete genome sequencing of *Lactobacillus pentosus* HP-B1718 and identification of the key enzyme for liquiritin biotransformation

**DOI:** 10.3389/fmicb.2026.1840719

**Published:** 2026-05-20

**Authors:** Zhi-Wen Tan, Xiang-Rui Mao, Qian-Meng Qi, Xiao-Han Wang, Meng-Jie Liu, Hang Wu, Lan-Fang Li, Zhao-Sen Fan, Shao-Yang Hou

**Affiliations:** 1 College of Pharmacy, Heze University, Heze, China; 2 Shandong Benon Biological Technology Co., Ltd., Heze, China

**Keywords:** biotransformation, complete genome, gene annotation, *Lactobacillus pentosus*, liquiritin

## Abstract

**Introduction:**

As a free aglycone with higher bioavailability and pharmacological activity, liquiritigenin can be obtained from liquiritin (liquiritigenin-4’-O-*β*-D-glucoside) catalyzed by Aryl-phospho-beta-D-glucosidase (designated as ApgA). Recent studies have revealed that the fermentation broth of *Lactobacillus pentosus* HP-B1718 can efficiently transform liquiritin into liquiritigenin.

**Methods:**

In this study, the complete genome of *Lactobacillus pentosus* HP-B1718 was completely sequenced using complete-genome sequencing technology. The results showed that the genome size of this strain was 3,257,491 bp with a GC content of 44.58%, presenting as a circular chromosome. It contained 3,081 CDS, 67 tRNAs, 1 tmRNA, and 16 rRNAs, together with a circular plasmid of 53,560 bp in size and 38.68% GC content. Furthermore, the probiotic properties, environmental stress tolerance and antibacterial potential of the strain were verified based on the complete genome data, comprehensively evaluating the application potential of HP-B1718 as a functional strain. Meanwhile, combined with genome annotation, the key gene *apgA* involved in the biotransformation of liquiritin was identified via BLAST analysis, and its expression vector was cloned and constructed.

**Result:**

The genome size of this strain was 3,257,491 bp with a GC content of 44.58%, presenting as a circular chromosome. It contained 3,081 CDS, 67 tRNAs, 1 tmRNA, and 16 rRNAs, together with a circular plasmid of 53,560 bp in size and 38.68% GC content. The purified ApgA with a molecular weight of 53.4 kDa was obtained through heterologous expression. Optimization of enzymatic reaction conditions demonstrated that the conversion rate of liquiritin reached 99% after incubating ApgA with 0.1% liquiritin for 4 h.

**Discussion:**

This study clarified the genomic characteristics of *Lactobacillus pentosus* HP-B1718 and the enzymatic properties of ApgA, provid¬ing a new theoretical basis and practical reference for the industrial production of liquiritigenin using ApgA.

## 1 Introduction

**Figure 1:**
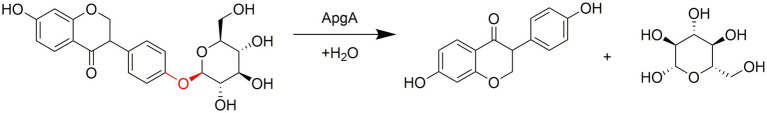
The reaction formula of Aryl-phospho-beta-D-glucosidase catalyzing the conversion of liquiritin to liquiritigenin. The glucosidic bond attacked by Aryl-phospho-beta-D-glucosidase is highlighted in red.

Licorice (Glycyrrhiza uralensis Fisch), also known as Mi Gan, Guo Lao and Mi Cao, has a long history of application in traditional Chinese medicine (Ikram et al., 2025). Licorice contains a variety of active components such as flavonoids, volatile oils and organic acids, and is widely used in biomedical related research due to its various excellent biological and pharmacological activities (Dang et al., 2024). Among all active components, liquiritin and liquiritigenin have attracted much attention because of their strong pharmacological activities (Wu et al., 2024). Liquiritin is a flavonoid glycoside formed by the conjugation of *β*-D-glucoside and liquiritigenin (Semwal et al., 2025) (Figure 1). Studies have shown that these two flavonoids have significant effects in anti-inflammation (Wu et al., 2022), cardiomyocyte injury (Mou et al., 2021), lung squamous cell carcinoma (Liu et al., 2024), anti-cardiovascular diseases (Markina et al., 2022), anti-neurodegenerative diseases (Ko et al., 2017), anti-microbial infection (Chen et al., 2024) and anti-diabetes (Liu et al., 2019).

Most naturally occurring licorice flavonoids exist in the form of glycosides with low biological activity (Wahab et al., 2021), The absorption of liquiritin by the human body is poor, and its aglycone form (liquiritigenin) can only be absorbed after hydrolysis by bacteria producing Aryl-phospho-beta-D-glucosidase in the intestinal tract or their enzymes (Sawadpongpan et al., 2023). Studies have shown that when used as a botanical drug, the physiological activity of liquiritigenin is usually stronger than that of liquiritin (Selyutina and Polyakov, 2019). For example, liquiritigenin has been proven to have stronger anti-glycosylation (Alvi et al., 2021), anti-free radical (Rasool and Dar, 2025) and anti-Aryl-phospho-beta-D-glucosidase activities (Semwal et al., 2025). Therefore, in pharmaceutical and food applications (Nascimento and de Araújo, 2022), it is necessary to convert liquiritin from glycoside-based components to aglycone-based components to improve human absorption rate and enhance pharmacological activity (Rasool and Dar, 2025). The traditional conversion method adopts acid hydrolysis, but this method has problems such as unsatisfactory conversion rate, production of harmful impurities and serious environmental pollution (Fan et al., 2016).

Compared with the traditional chemical hydrolysis method, the biotransformation of liquiritin into liquiritigenin using ApgA enzyme has become a research hotspot in recent years owing to its distinct advantages, including high reaction specificity, efficient conversion, and environmental friendliness (Hong et al., 2025). Previous studies have shown that the yield of liquiritigenin catalyzed by various commercially available high-purity enzymes derived from *Escherichia coli* is only 70–80% (Mittal et al., 2023). In addition, microorganisms such as *Aspergillus niger* (El-Far et al., 2024), *white-rot fungi* (Kijpornyongpan et al., 2022), and *Lactobacillus brevis* (Yuan et al., 2019) have also been investigated for the biosynthesis of liquiritigenin. However, most ApgA-producing microorganisms are potentially pathogenic or do not belong to generally recognized as safe (GRAS) strains (Finlay and Falkow, 1997). Therefore, the screening of food-grade microorganisms and the optimization of their enzyme production conditions remain important research directions in this field.

*Lactobacillus pentosus* is a heterofermentative lactic acid-producing bacterium that can be isolated from various environments such as human intestinal tract, vagina, oral cavity and fermented food (Stergiou et al., 2021). Strains of this genus have attracted wide attention due to their potential benefits to human health, so it is of great significance to study the flavonoid metabolic mechanism of *Lactobacillus pentosus* (Park et al., 2021). In this study, a strain of *Lactobacillus pentosus* HP-B1718 was found to be capable of converting liquiritin into liquiritigenin. Based on this finding, complete genome sequencing and annotation of HP-B1718 were performed. Combined with the complete genome data, the probiotic properties, environmental tolerance and antibacterial potential of the strain were verified to comprehensively evaluate its application value as a functional strain. Meanwhile, the enzymatic properties of the strain were characterized, the key enzyme involved in the conversion of liquiritin to liquiritigenin was identified, and its application potential in the industrial production of liquiritigenin was further explored.

## 2 Materials and methods

### 2.1 Strain culture conditions and plasmids

The strains and plasmids used in this study are shown in Table 1. *Lactobacillus pentosus* HP-B1718 was isolated from sufu in Guangxi Zhuang Autonomous Region, China, and the pure culture was preserved by anaerobic culture at 37 °C for 24 h in De Man-Rogosa-Sharpe (MRS) medium (BD, USA). Unless otherwise specified, all *Escherichia coli* strains were cultured at 37 °C in LB medium (HiMedia, India) containing 2% agar, and 50 μg/mL kanamycin was added when necessary. Liquiritin and liquiritigenin were purchased from Sigma (St. Louis, Missouri, USA), and other chemical reagents and solvents were of analytical grade and purchased from standard commercial channels.

**Table 1:** Strains and plasmids were used and constructed in this study.

Strains/plasmids	Description	Reference
Bacterial strains		
*Lactobacillus pentosus* HP-B1718	*Lactobacillus pentosus* HP-B1718 producing Aryl-phospho-*β*-D-glucosidase	This study
*Escherichia coli* BL21 (DE3)	Host strain for protein expression	invitrogen
*Escherichia coli* GB05-dir	Host strain for Red/ET-mediated recombination	invitrogen
Plasmids		
pET28a (+)	Expression vector	This study
pET28a (+)-*apgA*	Expression plasmid for Aryl-phospho-beta-D-glucosidase from HP-B1718	This study

### 2.2 Isolation, purification and phylogenetic analysis of *Lactobacillus pentosus* HP-B1718

Probiotic strains were isolated using the gradient dilution method. Briefly, 5 g of fermented bean curd was mixed with 45 mL of sterile physiological saline, shaken at 37 °C for 30 min, and centrifuged to collect the supernatant. Serial dilutions (10^−5^, 10^−6^, 10^−7^) were prepared, and 100 μL of each dilution was spread onto MRS agar medium (CM188, Beijing Bridge Technology Co, Ltd.). After incubation at 37 °C for 48 h, colony growth was observed. Strain HP-B1718 was identified by 16S rRNA gene sequencing, and the obtained sequence was aligned for homology analysis. Multiple sequence alignment was performed using the CLUSTAL X program, and a phylogenetic tree was constructed with the neighbor joining method in the MEGA software package (version 7.0.26). Pairwise distances for the neighbor joining tree were calculated using the Kimura 2 parameter model, and bootstrap analysis was conducted with 1,000 replicates.

### 2.3 Genomic DNA extraction and sequencing

Genomic DNA of *Lactobacillus pentosus* HP-B1718 was extracted using the HiPurA™ Bacterial Genomic DNA Purification Kit (Thermo Fisher Scientific, USA) (Ganesh et al., 2025) (whole-genome sequencing was completed through cooperation between our laboratory and a third-party company). The complete genomic sequence of HP-B1718 was obtained by nanopore sequencing and Illumina short-read sequencing:

Nanopore sequencing: The genomic DNA sequencing library was constructed according to the instructions of SQK-LSK109 Ligation Sequencing Kit and Native Barcoding Expansion Kit (Oxford Nanopore Technologies, UK), and the library was loaded on R9.4 sequencing chip for sequencing on PromethION sequencer (Oxford Nanopore Technologies, UK).
Illumina sequencing: 2 μg of genomic DNA was taken, and the sequencing library was constructed according to the instructions of NEBNext® Ultra™ DNA Library Prep Kit for Illumina® (New England Biolabs, USA) and sequenced on Illumina NovaSeq platform (Illumina, USA).

### 2.4 Genome annotation and analysis

Prodigal (v2.6.3), Aragorn (v1.2.38), RNAmmer (v1.2) and Infernal (v1.1) were used to predict the coding sequences (CDS), tRNA, rRNA and miscRNA in the sequenced genomic library, respectively. MinCED (v0.4.2), IslandViewer 4^1^ and PhiSpy^2^ were used to predict clustered regularly interspaced short palindromic repeats (CRISPRs), genomic islands and prophages, respectively.

Eight databases were used for functional annotation of the genome of *Lactobacillus pentosus* HP-B1718, including UniProt,^3^ KEGG,^4^ KEGG Pathway,^5^ GO,^6^ Pfam,^7^ COG,^8^ TIGERfams,^9^ RefSeq,^10^ and NR.^11^ BLAST+ (version: 2.11.0+) was used to compare the predicted gene sequences in *Lactobacillus pentosus* HP-B1718 with functional databases. Furthermore, ARDB^12^ database and CAZy^13^ database were used for further research on the functional annotation of the coding genes of *Lactobacillus pentosus* HP-B1718. Pathogen-host interaction genes, drug resistance genes, cytochrome P450, signal peptides, type III secretion system effectors and TCDB transporters were analyzed.

### 2.5 Thermotolerance assay of *Lactobacillus pentosus* HP-B1718

A single colony of *Lactobacillus pentosus* HP-B1718 was inoculated into MRS liquid medium and anaerobically cultured at 37 °C with 100 r/min for 24 h (Moon et al., 2023). The bacterial suspension was heated at 37 °C, 45 °C, and 50 °C for 1–3 min, respectively, diluted to 10^−6^ and 10^−7^ CFU/mL, and then spread on MRS solid medium. After anaerobic incubation at 37 °C for 20 h, the number of viable bacteria was counted (Roncarati et al., 2024).

### 2.6 Determination of acid tolerance and bile salt tolerance of *Lactobacillus pentosus* HP-B1718

*Preparation of Simulated Gastric Juice*: A total of 1.64 mL of dilute hydrochloric acid, 80 mL of distilled water, and 1 g of pepsin (Macklin, MFCD00081840, 3,000 U/mg) were thoroughly mixed. The mixture was adjusted to 100 mL with distilled water, and the pH was separately adjusted to 2.30, 3.24, and 4.31. The solution was filtered through a 0.44 μm membrane filter and stored at 4 °C until use (Zhou et al., 2022).

*Preparation of Simulated Intestinal Juice*: A total of 0.68 g of potassium dihydrogen phosphate was dissolved in 50 mL of distilled water, and the pH was adjusted to 6.8 with 0.1 mol/L sodium hydroxide. Then 0.5 g of trypsin (1:250, Macklin, MFCD00082094, ≥ 250 U/mg) was added and mixed thoroughly. The volume was adjusted to 100 mL with distilled water. Bovine bile salts (Macklin, B875069, ≥ 75.0%) were added to final concentrations of 0.03, 0.1, 0.2, and 0.3%, respectively. The solution was filtered through a 0.44 μm membrane filter and stored at 4 °C until use (Woelfel et al., 2024).

A 240 mL volume of HP-B1718 fermentation broth cultured for 20 h was centrifuged at 3580 r/min for 15 min. The supernatant was discarded, and the cell pellet was resuspended in 6 mL of physiological saline. This resuspended cell suspension without treatment in simulated gastrointestinal fluid served as the initial concentration control and was directly subjected to gradient dilution and plating, designated as the 0-min control group. In addition, 12 centrifuge tubes were divided into two groups: 6 tubes were added with 6 mL of simulated gastric juice, and the other 6 tubes with 6 mL of simulated intestinal fluid. A 200 μL aliquot of the above resuspended cell suspension was added to each tube and mixed thoroughly. After incubation for 150 min, the samples were serially diluted and plated onto MRS agar medium. Following incubation under appropriate conditions for 18–24 h, colony counting was performed, and the survival rate of the strain was calculated:


$$\text{Relative viable count ratio of}\;\mathrm{HP}-B1718\;(\%)=\frac{N}{N_{0}}\times 100\%$$

Where N represents the number of viable bacteria after treatment (CFU/mL), and N represents the initial number of viable bacteria (CFU/mL).

### 2.7 Determination of antibacterial activity of *Lactobacillus pentosus* HP-B1718 fermentation broth

Fourteen indicator strains were selected (all common pathogenic and drug-resistant strains used in this study were clinical isolates): *Salmonella typhi* HP-B1155, *Escherichia coli* HP-B1156, *Bacillus cereus* HP-B1157, *Klebsiella pneumoniae* HP-B1158, *Acinetobacter baumannii* HP-B1160, *Proteus mirabilis* HP-B1162, drug-resistant *E. coli* HP-B1163, drug-resistant *A. baumannii* HP-B1164, drug-resistant *Pseudomonas aeruginosa* HP-B1166, drug-resistant *Staphylococcus aureus* HP-B1167, *Shigella flexneri* HP-B1168, DH5α HP-B1169, BL21 HP-B1170, and *Shigella sonnei* HP-B1171. Indicator strains were inoculated into LB broth and incubated at 37 °C for 20 h before use. *Lactobacillus pentosus* HP-B1718 was inoculated into MRS broth and cultured at 37 °C for 24 h. The fermentation broth was centrifuged at 4500 r/min for 15 min, and the supernatant was filtered through a 0.22 μm microporous membrane. The antibacterial activity of the cell-free filtrate against indicator strains was determined using the Oxford cup method.

### 2.8 Cloning and sequence analysis of *apgA* gene of *Lactobacillus pentosus* HP-B1718

Genomic DNA of *Lactobacillus pentosus* HP-B1718 was extracted with reference to the previously reported method (Ye et al., 2020). The primers used in this study are shown in Table 2. According to the annotated sequence of *apgA* gene obtained by complete genome analysis, specific primer pair 1718-*apgA*-F/1718-*apgA*-R was designed to directly amplify the *apgA* gene encoding Aryl-phospho-beta-D-glucosidase from the genomic DNA of *Lactobacillus pentosus* HP-B1718. The amplified DNA fragment contained the coding region of *apgA* and the homology arm of the plasmid vector. Primers 28a-R/28a-R-1 and 28a-F/28a-F-1 were designed to amplify the vector pET28a fragment, respectively. The pET28a (+)-*apgA* expression plasmid was constructed by Red/ET cloning technology using GB05-dir recombinase and transformed into *Escherichia coli* BL21 (DE3) for heterologous expression. Universal primer pair T7/T7-term was used to amplify the cloned gene to confirm the completion of the target vector construction.

**Table 2:** Primers were used in this study.

Name	Sequence (5′-3′)	Purpose
1718-*apgA*-F	TGGTGCCGCGCGGCAGCCATATGGCTAGCATGACTGGTGGACAGCAAATGATGGATAAGGAGCGACAGATG	For cloning and expression of *apgA*
1718-*apgA*-R	TTAGCAGCCGGATCTCAGTGGTGGTGGTGGTGGTGCTCGAGTGCGGCCGCTTACAATTCATCTCCATTTTTTGCTAGG	
28a-F	CATTTGCTGTCCACCAGTCATGCTAG	For the construction of the pET28a (+)-*apgA* expression plasmid
28a-F-1	GAGGATGCTCACGATACGGGTTACTGATG	
28a-R	GCGGCCGCACTCGAGCACCACCAC	
28a-R-1	CGTTCCAGTAACCGGGCATGTTCATCATC	

### 2.9 Heterologous expression and purification of recombinant enzyme (ApgA)

Recombinant *Escherichia coli* BL21 was cultured at 37 °C until the absorbance at 600 nm (OD600) reached 0.6, then 0.1 mM isopropyl-*β*-D-thiogalactopyranoside (IPTG) was added, and the expression ApgA was induced at 16 °C for 24 h, after which the bacterial cells were collected.

bacterial cells were collected from 3 L of culture, resuspended in 70 mL of citrate buffer (pH 4.5), and disrupted by ultrasonic wave (Vibra Cell, Kosheng Ultrasonic, USA) with working for 4 s, pausing for 4 s, amplitude of 40% for 30 min. Insoluble lysate was removed by centrifuging the cell lysate at 12,500 rpm for 30 min at 4 °C to obtain crude enzyme solution. The crude enzyme solution was introduced into a Ni affinity chromatography column. The target protein was eluted with 20 mL of elution buffer consisting of 300 mmol/L NaCl, 50 mmol/L NaHPO₄, 160 mmol/L imidazole and 10 mmol/L Tris base at pH 7.4. To evaluate the purity of the eluted protein, 12% sodium dodecyl sulfate-polyacrylamide gel electrophoresis (SDS-PAGE) was used to analyze the purity of the purified enzyme, and the purified enzyme was stored at −80 °C in buffer containing 20% glycerol for later use. The protein concentration was determined by Bradford method with bovine serum albumin as the standard.

### 2.10 Biotransformation of liquiritin catalyzed by recombinant ApgA

The *in vitro* biotransformation experiment of liquiritin was carried out in a 250 mL shake flask system. The transformation conditions were set as follows: optimal reaction pH 4.5, temperature 38 °C, shaker rotation speed 100 r/min, and reaction time 12 h. The total volume of the reaction system was 25 mL, containing citric acid buffer with different concentrations of liquiritin and purified enzyme solution (10 U/mL), which served as the positive control. Meanwhile, three groups of negative controls were set up to exclude non-specific reactions and other interference factors: namely, the liquiritin buffer system without enzyme, the blank system containing only enzyme and buffer, and the liquiritin buffer system with boiled inactivated enzyme. After sampling at the set time points, the samples were extracted three times with an equal volume of ethyl acetate. The extract was concentrated by vacuum rotary evaporation and redissolved with 500 μL of methanol for high-performance liquid chromatography (HPLC) detection and analysis.

### 2.11 Quantification by HPLC external standard method

An appropriate amount of liquiritin standard was accurately weighed, dissolved in methanol, and diluted to a fixed volume to prepare a standard stock solution with a mass concentration of 100 mg/L. Subsequently, the stock solution was serially diluted with methanol to prepare a series of standard working solutions at different concentrations. Quantification by HPLC was performed using the external standard method, with the standard curve established using liquiritigenin standards at various concentrations. The conversion rate of liquiritin was calculated from substrate consumption according to the following equation.


$$\text{Liquiritin conversion rate}=\frac{C_{0}-C_{t}}{C_{0}}\times 100\%$$

where C_0_ is the initial mass concentration of liquiritin (mg/L), and C_t_ is the residual mass concentration of liquiritin in the system at the end of the reaction (mg/L). The result is expressed as a percentage (%).

### 2.12 HPLC and mass spectrometry condition

High-performance liquid chromatography (HPLC) analysis was performed on an Agilent 1,260 series system (Agilent Technologies, USA) equipped with an Agilent ODS-C18 reversed-phase column (5 μm, 4.6 × 250 mm) and a UV detector set at 280 nm. The mobile phase consisted of acetonitrile and 0.1% (v/v) acetic acid in water, with a total run time of 40 min. The injection volume was 10 μL, the flow rate was maintained at 1.0 mL/min, and the column temperature was set to 30 °C. For quantitative analysis, a liquiritin standard stock solution (0.5 mg/mL) was prepared in methanol, and a liquiritigenin standard stock solution (0.5 mg/mL) was prepared in acetonitrile-0.1% (v/v) acetic acid aqueous solution.

Following chromatographic separation, the eluates containing target compounds were collected and subjected to high-resolution time-of-flight mass spectrometry (TOF-MS). The analysis was performed using an electrospray ionization (ESI) source in negative ion mode (ESI-). The mass scan range was set according to the molecular weights of the target analytes. The mass tolerance for formula assignment was set to 5.0 mDa, with a degree of unsaturation (DBE) ranging from −1.5 to 50.0. The molecular formula was further validated using the isotopic distribution of three major isotope peaks.

### 2.13 Enzymatic characteristics of recombinant ApgA

Enzymatic activity of ApgA was determined by measuring its ability to hydrolyze the substrate liquiritin over a 30-min period at 38 °C and pH 4.5. The reaction mixture, with a total volume of 150 mL, contained 10 mg of ApgA and 10% (w/v) liquiritin. The reaction was terminated by adding an equal volume of ethyl acetate, and the product was analyzed by HPLC. One unit (1 U) of enzymatic activity was defined as the amount of enzyme required to hydrolyze 1 μmol of liquiritin per minute under the assay conditions. All data represent the mean values of three independent experiments.

To investigate the effects of pH and temperature on the activity of purified ApgA, the reaction system containing 50 mM citrate buffer (pH 4.5) was incubated at various temperatures to determine the optimal temperature, while other conditions remained constant. For optimal pH determination, the enzyme was dialyzed overnight at 4 °C in different 50 mM buffers (citrate buffer for pH 3.0–6.0, potassium phosphate buffer for pH 6.0–7.5, and Tris HCl buffer for pH 7.5–9.0), after which residual activity was measured. Thermostability was assessed by incubating the enzyme in 50 mM citrate buffer (pH 4.5) at various temperatures for 12 h, followed by measurement of residual activity. pH stability was determined by dialyzing the enzyme in various 50 mM buffers at 4 °C for 12 h and measuring the remaining activity. After determining the optimal pH and temperature for the enzyme, reaction rates at varying substrate concentrations were measured under these optimized conditions.

### 2.14 Optimization of liquiritin biotransformation mediated by purified ApgA

Meanwhile, the capacity of the purified enzyme to produce liquiritigenin was investigated under optimized conditions. Liquiritin at various concentrations (0.05, 0.1, 0.15, and 0.2%) was incubated with the purified enzyme, and the biotransformation efficiency was analyzed by HPLC. Two-way analysis of variance (Two-way ANOVA) was performed using SPSS 26.0 software to evaluate the effects of substrate concentration (factor a), reaction time (factor b), and their interaction on the yield of liquiritigenin.

## 3 Results

### 3.1 Morphological observation and phylogenetic analysis of *Lactobacillus pentosus* HP-B1718

**Figure 2:**
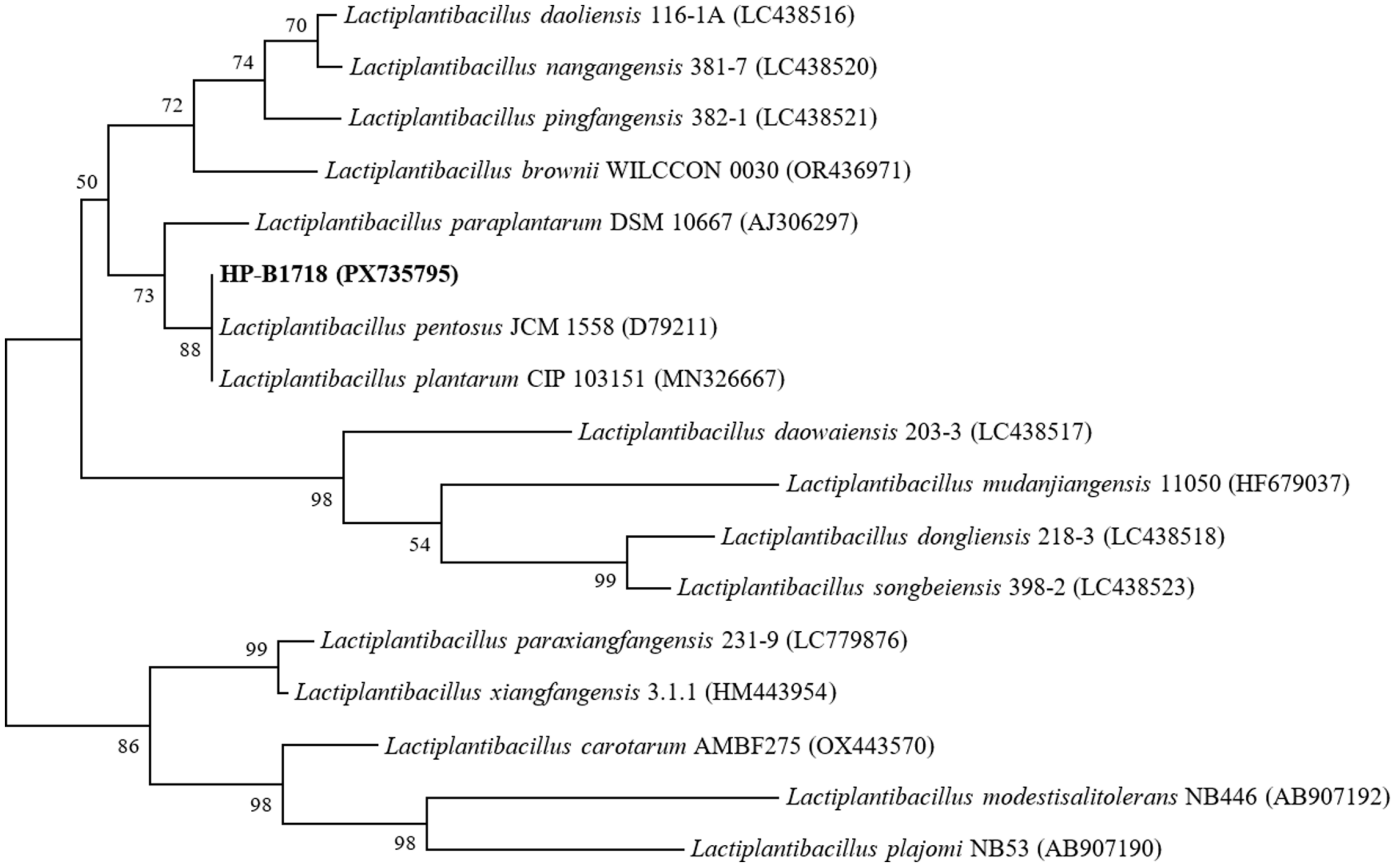
Phylogenetic tree constructed based on 16S rRNA gene sequence by neighbor-joining method, showing the phylogenetic relationship between *Lactobacillus pentosus* HP-B1718 and closely related species.

Strain *Lactobacillus pentosus* HP-B1718 was isolated from fermented bean curd using the gradient dilution method. After cultivation on MRS agar, the colonies were approximately 3 mm in diameter, raised, circular, smooth, and finely textured, with a light to deep yellow color. The strain was Gram-positive, non-spore-forming, and rod-shaped. The purified strain HP-B1718 has been deposited in the China Center for Type Culture Collection (CCTCC) under the accession number CCTCC NO: M20252617. The 16S rRNA gene sequence of HP-B1718 has been submitted to the NCBI GenBank database under the accession number PX735795.1 (available at https://www.ncbi.nlm.nih.gov/nuccore/PX735795.1/). A phylogenetic tree based on the 16S rRNA gene sequences was constructed using the neighbor-joining method (Figure 2).

### 3.2 Genomic characteristics of *Lactobacillus pentosus* HP-B1718

**Figure 3:**
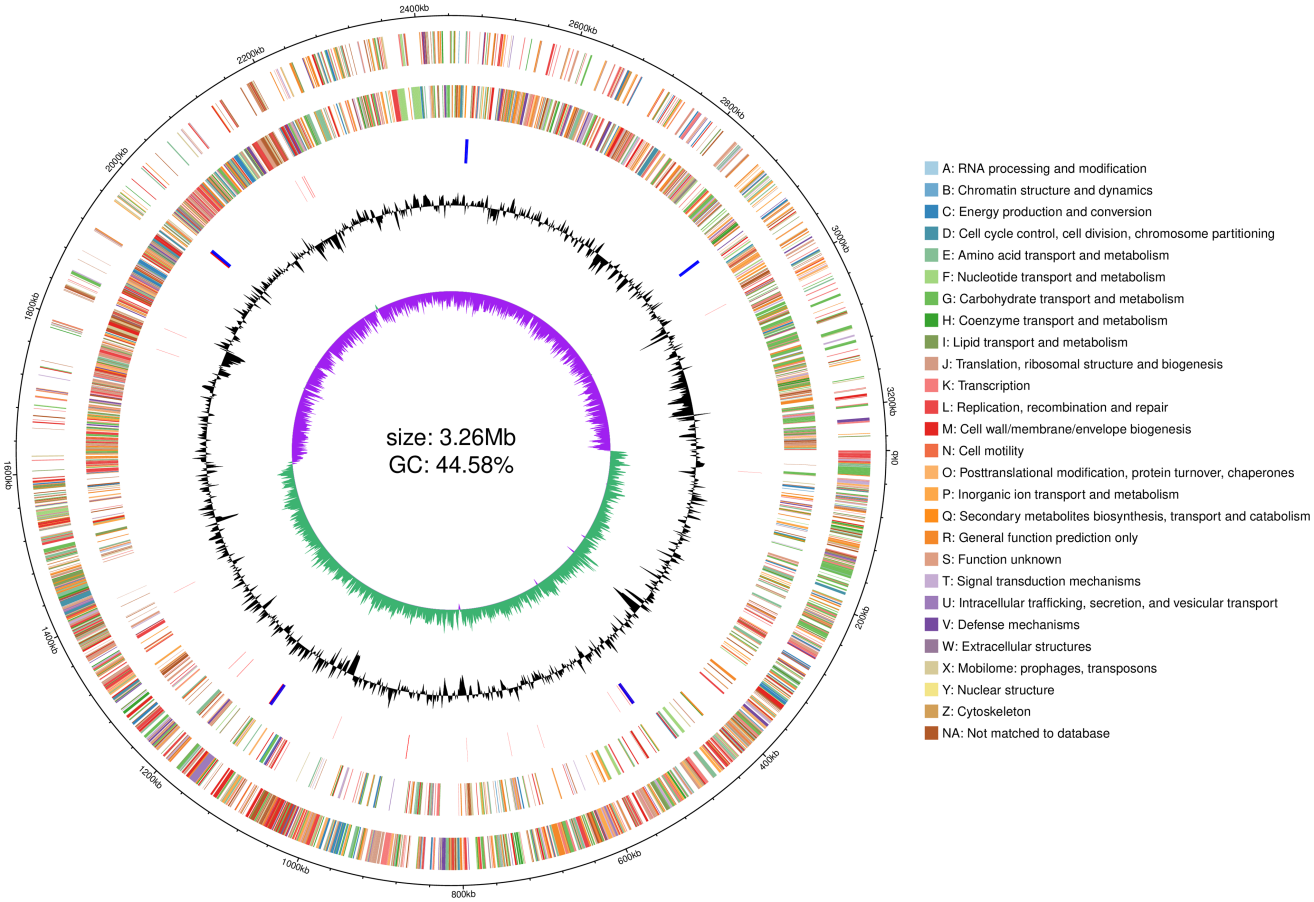
Circular genome map of *Lactobacillus pentosus* HP-B1718. From outside to inside: the first circle, genome coordinates. The second circle, genes on the positive strand of the genome sequence, with different colors representing different COG functional classifications. The third circle, genes on the negative strand of the genome sequence, with different colors representing different COG functional classifications. The fourth circle, rRNA and tRNA in the genome sequence, rRNA in blue and tRNA in red. The fifth circle, G + C content curve with a 2,000 bp sliding window. The sixth circle, G + C skew curve with a 2,000 bp sliding window.

The complete genome sequence of *Lactobacillus pentosus* HP-B1718 was obtained and assembled using Nanopore and Illumina sequencing technologies. The genomic map of this strain is shown in Figure 3. The genome is a circular molecule of 3,257,491 bp with a GC content of 44.58%, containing 3,081 CDSs, 67 tRNAs, 1 tmRNA, 16 rRNAs, and 1 plasmid (Supplementary Figure 1). The plasmid is also circular, with a size of 53,560 bp and a GC content of 38.68%. The genome contains 11 CRISPR sequences, 5 genomic islands, 10 prophages, 162 repeat sequences (total length: 12,248 bp), and 4,189 promoters. The draft genome sequence of *Lactobacillus pentosus* HP-B1718 has been deposited in the NCBI GenBank database under the accession number JBXEEH000000000.

### 3.3 Genome annotation and functional analysis of *Lactobacillus pentosus* HP-B1718

In this study, functional annotation of the predicted gene sequences was summarized using eight major databases, and the annotation statistics are presented in Supplementary Table 1. Among these, 2,425 genes (80.81%) were annotated in the Pfam database, and the top 20 protein domains ranked by annotation count were visualized in Supplementary Figure 2. Gene functions were predicted and classified by alignment against the Unigene and COG databases to reveal the global distribution pattern of gene functions in this strain. A total of 2,540 genes (82.44%) were assigned to 26 functional categories, and the COG classification of annotated genes is shown in Supplementary Figure 3.

In KEGG pathway analysis, 2,336 genes (75.82%) were functionally annotated, among which 1,160 genes (37.65%) were mapped to specific metabolic pathways. As a major public database for systematic analysis of intracellular metabolic pathways and functions of gene products, KEGG facilitates in-depth exploration of the complex biological behaviors of genes. In this study, KEGG-annotated genes were categorized according to the metabolic pathways in which they participate, and the results are displayed in Supplementary Figure 4.

Furthermore, 2,348 genes (76.21%) were annotated in the Gene Ontology (GO) analysis. As an internationally standardized classification system for gene function, GO employs a dynamically updated controlled vocabulary to comprehensively describe the properties of genes and their products at three levels: biological process, cellular component, and molecular function. Based on the GO database, target protein sequences were functionally annotated via BLAST alignment, as shown in Supplementary Figure 5.

**Figure 4:**
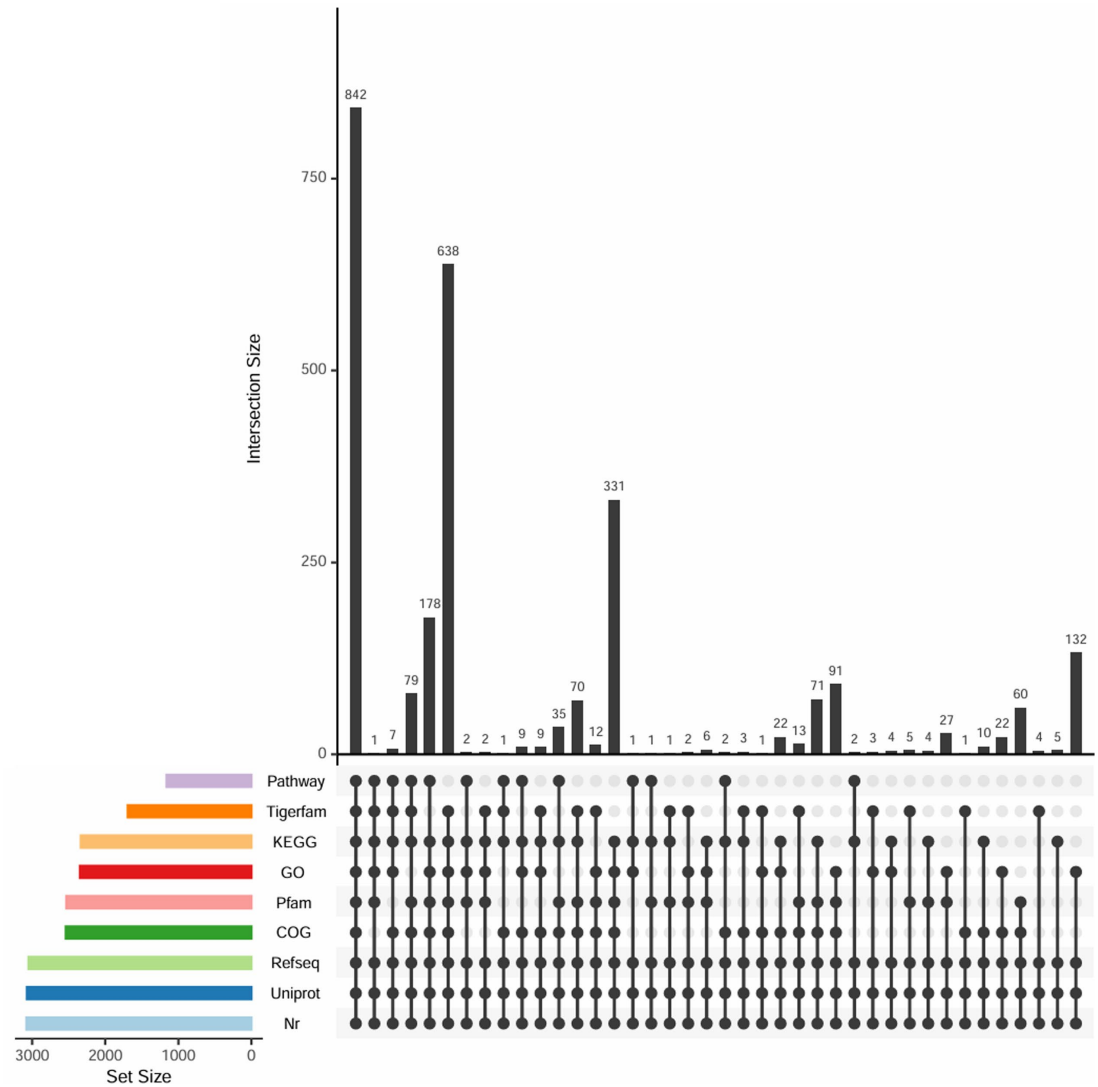
Common and unique gene annotations of *Lactobacillus pentosus* HP-B1718 in different databases.

Among other databases, 3,073 genes (99.74%) were functionally annotated in the Uniprot database, 3,051 genes (99.03%) in the Refseq database, and 3,080 genes (99.97%) in the non-redundant (NR) protein database. The NR database integrates non-redundant sequences from SwissProt, PIR, PRF, and PDB, as well as protein sequences translated from coding sequences in GenBank and RefSeq. In this study, target protein sequences were annotated against the NR database using Diamond BLASTp with parameters: -evalue 1e-5, −max_target_seqs 1, and the results are shown in Supplementary Figure 6. Meanwhile, 1,648 genes (54.92%) were annotated in the Tigrfams database. The shared and unique annotations among different databases are summarized in Figure 4.

By comparison against the ARDB and CAZy databases, the following annotation results were obtained: no resistance genes homologous to the target sequences were detected in the ARDB database; five classes of functional enzymes were annotated in the CAZy database; 1,295 full-length gene sequences showing 100% identity with the target sequences were retrieved from the PHI database; and two drug resistance-related genes were identified in the CARD database. In addition, a total of 212 signal peptides, 885 transmembrane proteins, and 93 secreted proteins were predicted. Within the genome of *Lactobacillus pentosus* HP-B1718, 293 gene sequences with 100% homology to the target gene sequences were annotated as type III secretion system effector proteins. The annotation results of TCDB membrane transporters are detailed in Supplementary Figure 7.

Genome-wide analysis of *L. pentosus* HP-B1718 systematically revealed the functional characteristics of its predicted genes, laying a foundation for further in-depth research on this strain. The functional potential of this strain holds promising application value in biotechnology, including drug development and biocatalytic transformation. Given the high species diversity of *L. pentosus*, different strains within this species and different species within the genus may exhibit distinct strain-specific and species-specific phenotypes, providing valuable insights for evolutionary studies at the species and genus levels.

### 3.4 Thermal tolerance of *Lactobacillus pentosus* HP-B1718

The survival rate assay showed that a large number of viable *Lactobacillus pentosus* HP-B1718 cells remained after heating at 50 °C for 3 min, with a viable count of 4.8 × 10^8^ CFU/mL and a survival rate of 13.11%, indicating that *Lactobacillus pentosus* HP-B1718 exhibited favorable thermal tolerance. The results are presented in Supplementary Table 2.

### 3.5 Acid tolerance and bile salt tolerance of *Lactobacillus pentosus* HP-B1718

The results of the relative viable count ratio assay demonstrated that HP-B1718 exhibited excellent gastrointestinal tolerance. After incubation in simulated gastric juice at pH 2.30 for 150 min, the strain remained highly viable, with a viable cell count of 3.41 × 10^9^ CFU/mL and a relative viable count ratio of up to 331.06% (Davoren et al., 2018) (Supplementary Table 3), indicating strong acid tolerance. Following treatment in simulated intestinal juice containing 0.3% bile salts for 150 min, the viable cell count was 2.98 × 10^9^ CFU/mL and the relative viable count ratio was 151.26% (Khoo et al., 2025) (Supplementary Table 4), also revealing favorable bile salt resistance. The observation that the relative viable count ratio exceeded 100% was not attributed to significant proliferation of the strain in the simulated gastrointestinal fluids. Although the simulated gastrointestinal fluids imposed low pH and bile salt stress, they still contained trace amounts of basic nutrients such as carbon and nitrogen sources, allowing HP-B1718 to maintain fundamental metabolism without substantial cell death. Meanwhile, stress exposure activated the bacterial defense and metabolic systems, enabling rapid recovery of viability and transient proliferation during the subsequent plate resuscitation culture. Consequently, the counted colony number exceeded the initial inoculum level, resulting in a relative viable count ratio greater than 100%.

### 3.6 Antibacterial activity of *Lactobacillus pentosus* HP-B1718 fermentation broth

Antibacterial assay results showed that both the fermentation broth and its centrifuged supernatant of HP-B1718 exhibited strong inhibitory activity against pathogenic bacteria including *Salmonella typhi* HP-B1155, *Escherichia coli* HP-B1156, *Bacillus cereus* HP-B1157, *Klebsiella pneumoniae* HP-B1158, *Acinetobacter baumannii* HP-B1160, *Proteus mirabilis* HP-B1162, *drug-resistant E. coli* HP-B1163, *drug-resistant A. baumannii* HP-B1164, *drug-resistant Pseudomonas aeruginosa* HP-B1166, *drug-resistant Staphylococcus aureus* HP-B1167, *Shigella flexneri* HP-B1168, *DH5α* HP-B1169, *BL21* HP-B1170, and *Shigella sonnei* HP-B1171. The results determined by the Oxford cup method are shown in Supplementary Table 5, and the inhibition zone diameters are presented in Supplementary Figure 8. In most cases, the inhibition zone diameters exceeded 15 mm, and the diameter against *A. baumannii* was greater than 20 mm. Antibacterial tests demonstrated that both the fermentation broth and supernatant of HP-B1718 could inhibit the growth of various pathogenic bacteria.

### 3.7 Heterologous expression and purification of recombinant ApgA

**Figure 5:**
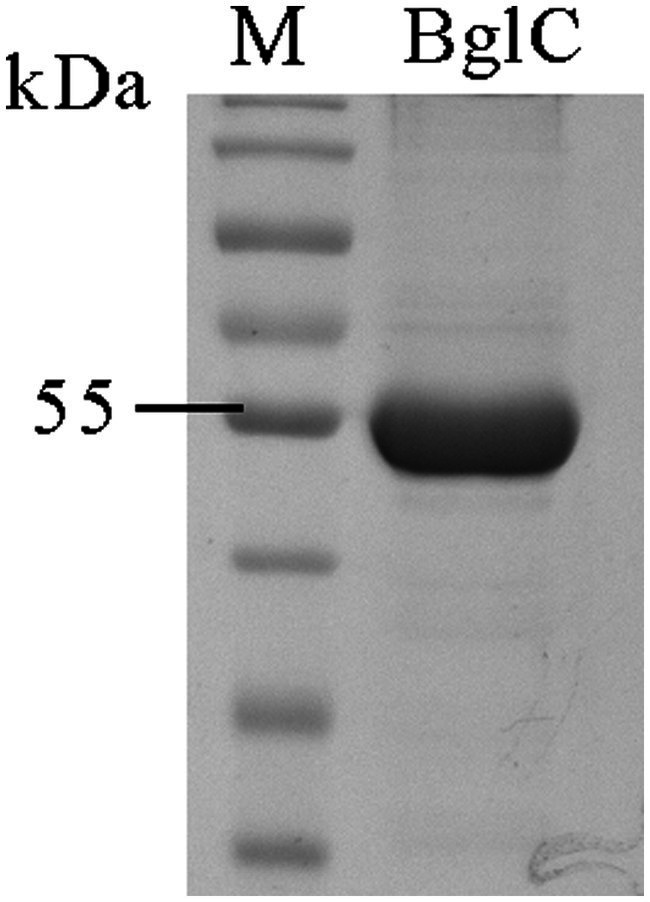
Sodium dodecyl sulfate-polyacrylamide gel electrophoresis (SDS-PAGE) analysis of purified protein. The figure shows a 12% SDS-polyacrylamide gel stained with Coomassie brilliant blue. Lane M is the molecular weight standard (Thermo, Cat. No. 26616).

The nucleotide sequence of the *apgA* gene encoding the recombinant ApgA cloned and expressed in this study has been deposited in the GenBank database under the accession number PZ286098. SDS-PAGE analysis showed that the purified recombinant enzyme presented a single band with a molecular weight of about 53.4 kDa, indicating that most of the hetero-proteins had been removed (Figure 5).

### 3.8 Construction of HPLC standard curve

Linear regression analysis was performed based on the contents and corresponding peak areas of the liquiritin standard to construct the standard curve (Supplementary Table 6), and the regression equation was obtained (Supplementary Figure 9): Y = 16.25X–26.57 (R^2^ = 0.9997), where Y represents the peak area and X stands for the mass concentration of liquiritin (mg/L). All enzymatic reactions were carried out in triplicate (n = 3), and the results are presented as mean ± standard deviation (SD) (Supplementary Table 7). The standard curve of liquiritin established in this study exhibited an excellent linear relationship with an R^2^ value higher than 0.999, indicating a strong linear correlation between peak area and liquiritin concentration within the tested concentration range. Therefore, this method can be employed for the accurate quantitative analysis of liquiritin in subsequent samples.

### 3.9 HPLC analysis and mass spectrometric identification of enzymatic transformation products

**Figure 6:**
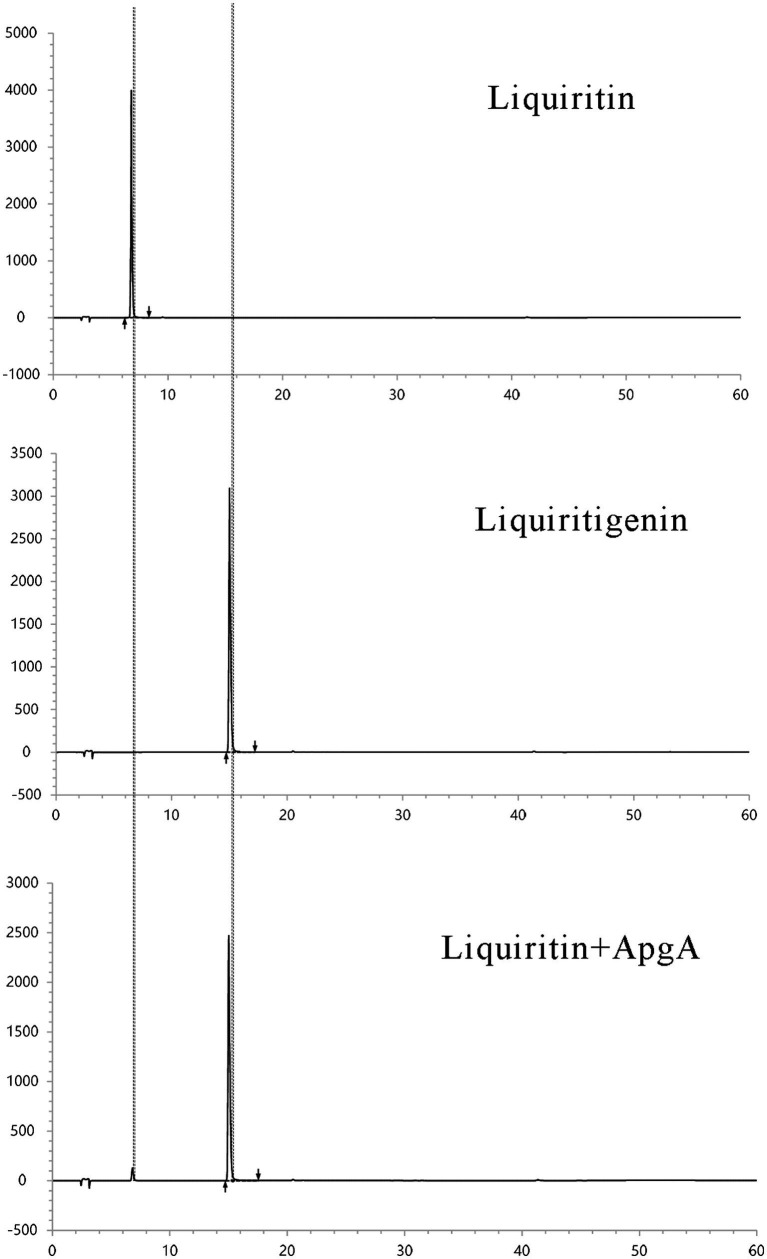
HPLC analysis results of liquiritigenin catalyzed by purified ApgA enzyme from *Lactobacillus pentosus* HP-B1718.

HPLC analysis of the enzymatic conversion reaction (Figure 6) was further confirmed by TOF-MS in negative ion mode (ESI^−^). The mass spectrum of the liquiritigenin standard (Supplementary Figure 10A) showed a quasi-molecular ion peak at m/z 255.0667 [M-H]^−^, which was highly consistent with the calculated value for the theoretical formula C₁₅H₁₁O₄^−^ (m/z 255.0657), with a mass error of only 3.9 ppm. This confirmed the molecular formula of liquiritigenin as C₁₅H₁₂O₄. For the liquiritin standard (Supplementary Figure 10B), the quasi-molecular ion peak appeared at m/z 417.1192 [M-H]^−^, matching the calculated value for C₂₁H₂₁O₉^−^ (m/z 417.1186) with a mass error of 1.4 ppm, verifying its molecular formula as C₂₁H₂₂O₉. In the reaction mixture of liquiritin and ApgA (Supplementary Figure 10C), both the substrate and product were detected. The quasi-molecular ion peak corresponding to liquiritin was observed at m/z 417.1183 [M-H]^−^, while the peak corresponding to the product liquiritigenin was detected at m/z 255.0656 [M-H]^−^. The simultaneous presence of these two ions confirmed the conversion of liquiritin to liquiritigenin catalyzed by ApgA.

### 3.10 Enzymatic characteristics of recombinant ApgA

The optimal temperature of recombinant ApgA was 40 °C (Supplementary Figure 11A), the enzyme activity loss was less than 25% in the range of 30–45 °C, and the enzyme activity decreased to about 90% of the maximum value at 35 °C. The enzyme was sensitive to temperature, only about 30% of the activity was retained after incubation at 60 °C for 12 h, and the activity was less than 20% at 65 °C (Supplementary Figure 11B). The optimal pH value was 4.5 (Supplementary Figure 11C), the enzyme activity loss was less than 20% in the range of pH 4.5–5.0, and the activity decreased to 60 and 20% of the maximum value at pH 3.0 and pH 7.0, respectively. The enzyme had good pH stability, and the enzyme activity loss was less than 10% after dialysis in the range of pH 4.2–7.2 for 12 h (Supplementary Figure 11D).

### 3.11 Optimization of liquiritin biotransformation mediated by purified ApgA

**Figure 7:**
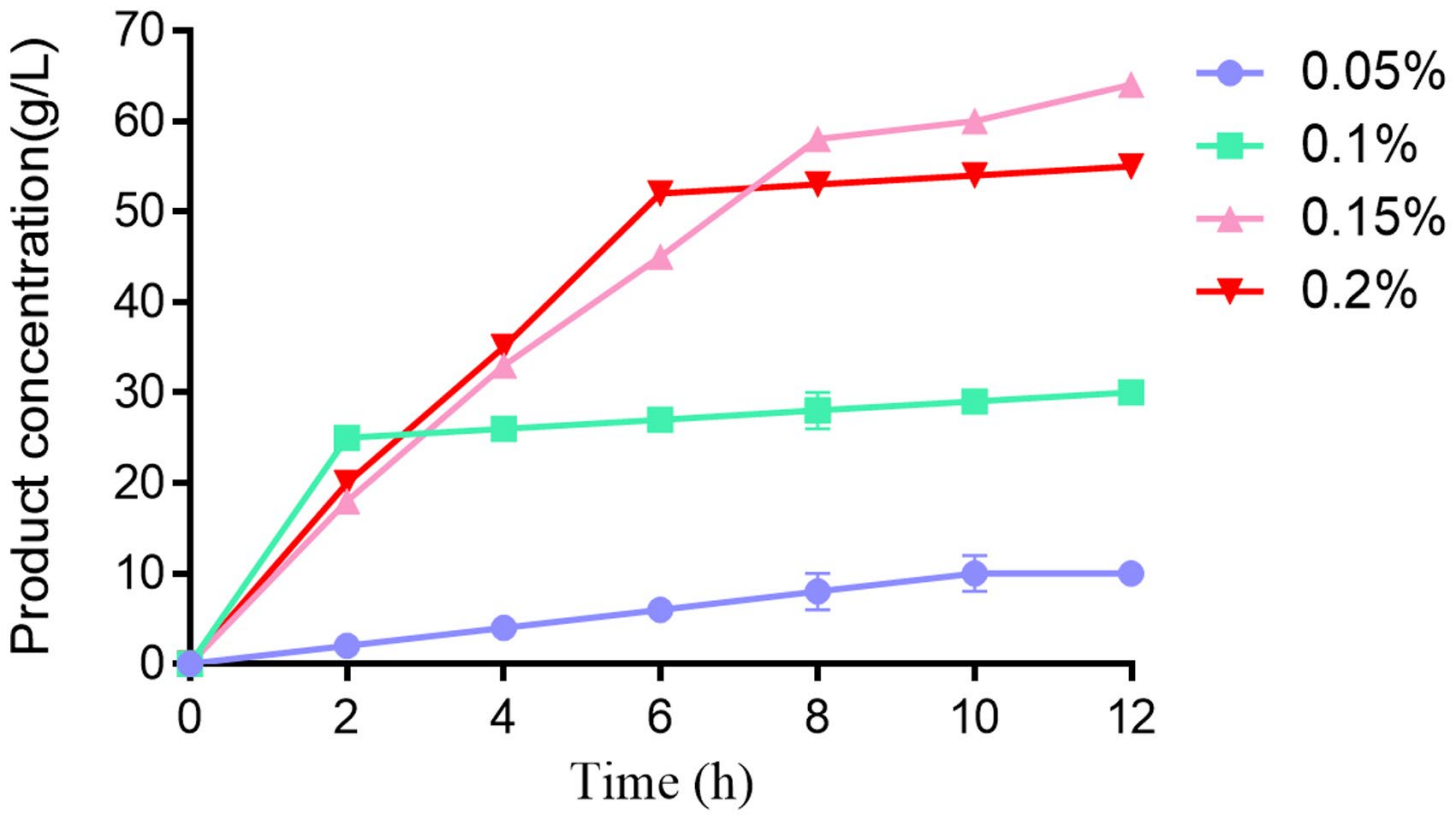
Time course curve of liquiritigenin production catalyzed by purified ApgA enzyme from *Lactobacillus pentosus* HP-B1718 under different liquiritin concentrations. The experiment was performed with three independent replicates.

The results of the incubation assay of liquiritin with the purified enzyme ApgA showed that ApgA exhibited remarkable catalytic conversion capability toward liquiritin *in vitro*, which could efficiently transform liquiritin into liquiritigenin. However, high substrate concentrations exerted a significant inhibitory effect on the catalytic activity of this enzyme (Figure 7). Specifically, when the initial liquiritin concentration was 0.2%, the yield of liquiritigenin reached a maximum of 765.2 mg/L after 10–12 h of incubation, with a corresponding conversion rate of 83%. In contrast, at an initial liquiritin concentration of 0.1%, a liquiritigenin yield of 606.37 mg/L was achieved after only 4 h of incubation, corresponding to an extremely high conversion rate of 99%. This yield was not only significantly higher than that of the 0.05% liquiritin group at the same incubation time point, but the time required for liquiritigenin production to peak was also 2 h earlier than that in the 0.05% group. In addition, high substrate concentrations markedly restricted the enzymatic reaction rate. At a liquiritin concentration of 0.2%, the time required for liquiritigenin production to peak was delayed to 6 h, further confirming the inhibitory effect of high substrate concentrations on the catalytic activity of ApgA.

To further verify the effects of substrate concentration and reaction time on liquiritigenin yield, as well as their interaction, two-way analysis of variance (two-way ANOVA) was performed on the experimental data. The results showed that substrate concentration (factor a) significantly influenced liquiritigenin yield (*F* = 81.068, *p* < 0.05), and reaction time (factor b) also significantly influenced liquiritigenin yield (*F* = 28.379, p < 0.05). Meanwhile, the interaction between substrate concentration and reaction time (a*b) significantly influenced liquiritigenin yield (*F* = 2.008, *p* = 0.035 < 0.05).

The ultra-high conversion rate of 99% achieved in this study resulted from the synergistic effects of multiple factors. On the one hand, the reaction system was optimized to the optimal pH (Gu, 2024) of 4.5 and temperature (Peterson et al., 2007) of 38 °C, providing a suitable environment for the enzyme to exert its catalytic activity. On the other hand, an appropriate enzyme dosage (Sahin et al., 2023) (10 U/mL) ensured sufficient catalytic sites, enabling rapid conversion of the substrate. More importantly, the substrate concentration of 0.1% fell within the suitable range for the enzyme, which not only avoided limited product formation caused by insufficient substrate but also did not induce the inhibitory effect caused by high substrate concentrations, allowing the enzymatic reaction to proceed nearly to completion.

## 4 Discussion

In this study, a strain of *Lactobacillus pentosus* HP-B1718 was found to be capable of converting liquiritin into liquiritigenin. Based on this finding, complete genome sequencing and annotation of HP-B1718 were conducted, and combined with the complete genome data, the probiotic properties, environmental tolerance and antibacterial potential of the strain were verified to comprehensively evaluate its application value as a functional strain. Meanwhile, the enzymatic properties of the strain were characterized, the key enzyme involved in the conversion of liquiritin to liquiritigenin was identified, and its application potential in the industrial production of liquiritigenin was further explored. *Lactobacillus pentosus* strains are considered generally recognized as safe (GRAS) microorganisms (Segers and Lebeer, 2014), and the Aryl-phospho-beta-D-glucosidase (ApgA) derived from them can serve as an effective tool for the safe biotransformation strategy of prodrugs such as liquiritin (Magwaza et al., 2024). In this study, the *apgA*-encoding gene of *Lactobacillus pentosus* HP-B1718 was sequenced, and heterologous expression and enzymatic characterization analysis were carried out to reveal the application efficiency of an unreported biocatalyst in liquiritin conversion.

The biotransformation of liquiritin to liquiritigenin is of great strategic significance in cancer prevention and treatment (Han et al., 2024), pathogenic bacterial infection and inflammation treatment. In this study (Sun et al., 2018), the purified Aryl-phospho-beta-D-glucosidase could completely convert 0.15% liquiritin within 6 h, and the conversion rate of 0.1% liquiritin reached 99%. Compared with the biotransformation efficiency (70–90%) (Setlow et al., 2004) of microbial Aryl-phospho-beta-D-glucosidase reported in existing literatures, the conversion rate in this study was increased by about 10%. The high conversion rate may be due to theinherent characteristics of Aryl-phospho-beta-D-glucosidase (Robinson, 2015), acidic reaction system and higher reaction temperature: the weakly acidic reaction environment is conducive to the protonation of substrate molecules and promotes the dissociation of *β*-D-glucuronic acid residues in liquiritin (Girame et al., 2022), and the protonated state will affect the interaction between substrate and enzyme protein, thereby improving the conversion efficiency of the enzyme to the substrate (Zapata-Torres et al., 2012). the pKa1 of liquiritin is 7.70, which can be completely protonated under weakly acidic conditions (Joos et al., 2024), and its stability is the best at pH 4.28, which is close to the reaction pH in this study (Stoffel et al., 2025). in addition, the higher reaction temperature helps to improve the solubility of poorly soluble liquiritin (Black and Muller, 2010), thus promoting biotransformation.

However, the high reaction temperature is a double-edged sword forthe enzymatic reaction. Although it can improve the substrate solubility, it will lead to the decrease of enzyme stability and limit the conversion efficiency of liquiritigenin (Mariñas-Collado et al., 2019). Enzyme stability analysis showed that the enzyme activity loss was more than half after 12 h (Vanella et al., 2024), and there was a slight activity loss even under the optimal pH condition (Hou et al., 2023). In addition, the increase of substrate concentration will lead to the decrease of conversion rate and enzyme activity. Existing studies have confirmed that the reaction by-product *β*-D-glucose can act as an inhibitor to limit enzymatic conversion (Bitter et al., 2023). Although high substrate concentration will cause enzyme inhibition, the characteristic that Aryl-phospho-beta-D-glucosidase can efficiently convert 0.1% liquiritin is completely suitable for the production of liquiritigenin (El-Far et al., 2024). In summary, this study deeply explored the characteristics of Aryl-phospho-beta-D-glucosidase derived from *Lactobacillus pentosus*, confirmed that it can catalyze the production of liquiritigenin with stronger pharmacological activity, and provided new ideas for the development of biological tools.

## Data Availability

The datasets presented in this study can be found in online repositories. The names of the repository/repositories and accession number(s) can be found at: https://www.ncbi.nlm.nih.gov/genbank/, PX735795.1.

## Footnotes

1
http://www.pathogenomics.sfu.ca/islandviewer/

2
https://github.com/linsalrob/PhiSpy

3
https://www.uniprot.org/

4
https://www.genome.jp/kegg/

5
https://www.genome.jp/kegg/pathway.html

6
https://www.geneontology.org/

7
https://www.ebi.ac.uk/interpro/entry/pfam/

8
https://ngdc.cncb.ac.cn/databasecommons/database/id/37

9
https://tigrfams.jcvi.org/cgi-bin/index.cgi

10
https://www.ncbi.nlm.nih.gov/refseq/

11
https://www.ncbi.nlm.nih.gov/protein/

12
https://ardb.cbcb.umd.edu/

13
https://www.cazy.org/

## References

[ref1] Alvi S. S. Nabi R. Khan M. S. Akhter F. Ahmad S. Khan M. S. (2021). Glycyrrhizic acid scavenges reactive carbonyl species and attenuates glycation-induced multiple protein modification: an In vitro and In silico study. Oxidative Med. Cell. Longev. 2021:7086951. doi: 10.1155/2021/7086951 PMC8548169 34712386

[ref2] Bitter J. Pfeiffer M. Borg A. J. E. Kuhlmann K. Pavkov-Keller T. Sánchez-Murcia P. A. . (2023). Enzymatic β-elimination in natural product O- and C-glycoside deglycosylation. Nat. Commun. 14:7123. doi: 10.1038/s41467-023-42750-0, 37932298 PMC10628242

[ref3] Black S. Muller F. (2010). On the effect of temperature on aqueous solubility of organic solids. Organic Process Res. Dev. 14, 661–665. doi: 10.1021/op100006y

[ref4] Chen R. Shi J. Liu Y. Yu J. Li C. Tao F. . (2024). The state-of-the-art antibacterial activities of glycyrrhizin: A comprehensive review. Microorganisms 12:1155. doi: 10.3390/microorganisms12061155, 38930536 PMC11206003

[ref5] Dang L. Jin Y. Yuan Y. Shao R. Wang Y. (2024). Licorice: comprehensive review of its chemical composition, pharmacodynamics, and medicinal value. Acupunct. Herbal Med. 4, 136–150. doi: 10.1097/HM9.0000000000000103

[ref6] Davoren M. J. Liu J. Castellanos J. Rodríguez-Malavé N. I. Schiestl R. H. (2018). A novel probiotic, *Lactobacillus johnsonii* 456, resists acid and can persist in the human gut beyond the initial ingestion period. Gut Microbes 10, 458–480. doi: 10.1080/19490976.2018.1547612, 30580660 PMC6748577

[ref7] El-Far S. W. Al-Saman M. A. Abou-Elazm F. I. Shebl R. I. Abdella A. (2024). Antimicrobial and antioxidant activities of 18β-Glycyrrhetinic acid biotransformed by *Aspergillus niger*. Microbiol. Res. 15, 1993–2006. doi: 10.3390/microbiolres15040133

[ref8] Fan R. Li N. Xu H. Xiang J. Wang L. Gao Y. (2016). The mechanism of hydrothermal hydrolysis for glycyrrhizic acid into glycyrrhetinic acid and glycyrrhetinic acid 3- O -mono-β- d -glucuronide in subcritical water. Food Chem. 190, 912–921. doi: 10.1016/j.foodchem.2015.06.039 26213056

[ref9] Finlay B. B. Falkow S. (1997). Common themes in microbial pathogenicity revisited. Microbiol. Mol. Biol. Rev. 61, 136–169. doi: 10.1128/mmbr.61.2.136-169.1997, 9184008 PMC232605

[ref10] Ganesh K. R. Ningaraju T. M. Peter A. Lakshminarayana Reddy C. N. Kavan Kumar V. (2025). Molecular cloning and heterologous expression of lipase gene from *Pseudomonas aeruginosa* in *Escherichia coli*. Int. J. Biol. Macromol. 297:139866. doi: 10.1016/j.ijbiomac.2025.139866 39818390

[ref11] Girame H. Garcia-Borràs M. Feixas F. (2022). Changes in protonation states of In-pathway residues can Alter ligand binding pathways obtained from spontaneous binding molecular dynamics simulations. Front. Mol. Biosci. 9:922361. doi: 10.3389/fmolb.2022.922361 35860361 PMC9289141

[ref12] Gu Y. (2024). The effect of buffer pH on enzyme activity. Theor. Nat. Sci. 33, 137–147. doi: 10.54254/2753-8818/33/20240893

[ref13] Han Y. Sheng W. Liu X. Liu H. Jia X. Li H. . (2024). Glycyrrhizin ameliorates colorectal cancer progression by regulating Nhej pathway through inhibiting Hmgb1-induced Dna damage response. Sci. Rep. 14:24948. doi: 10.1038/s41598-024-76155-w, 39438689 PMC11496679

[ref14] Hong S. Kim D. Kim S. Lee C. Lee S. Lee S. . (2025). Biotransformation by beta glucosidase enhances anti inflammatory metabolites in licorice using untargeted metabolomics. npj Sci. Food 9:533. doi: 10.1038/s41538-025-00533-5 PMC12325765 40764312

[ref15] Hou Q. Rooman M. Pucci F. (2023). Enzyme stability-activity trade-off: new insights from protein stability weaknesses and evolutionary conservation. J. Chem. Theory Comput. 19, 3664–3671. doi: 10.1021/acs.jctc.3c00036, 37276063

[ref16] Ikram A. Shahzadi M. Tayyab H. Awlqadr F. H. Arshad M. T. Parveen H. . (2025). Licorice: a review of nutritional, medicinal, economic, and toxicity aspects. Int. J. Food Sci. Technol. 60:236. doi: 10.1093/ijfood/vvaf236

[ref17] Joos M. Vackier T. Mees M. A. Coppola G. Alexandris S. Geunes R. . (2024). Antimicrobial activity of Glycyrrhizinic acid is pH-dependent. Acs Appl. Bio Mater. 7, 8223–8235. doi: 10.1021/acsabm.4c00942, 39592134 PMC11655076

[ref18] Khoo S. C. Chin K. W. Ting T. Z. Luang-In V. Lan J. C. Ma N. L. (2025). Stress tolerance and metabolism profiling of selected functional probiotic strains. Food Biosci. 64:105919. doi: 10.1016/j.fbio.2025.105919

[ref19] Kijpornyongpan T. Schwartz A. Yaguchi A. Davinia S. (2022). Systems biology-guided understanding of white-rot fungi for biotechnological applications: a review. Cell Press 25:104640. doi: 10.1016/j.isci.2022.104640, 35832889 PMC9272384

[ref20] Ko Y. Kwon S. Lee S. Jang C. (2017). Liquiritigenin ameliorates memory and cognitive impairment through cholinergic and Bdnf pathways in the mouse hippocampus. Arch. Pharm. Res. 40, 1209–1217. doi: 10.1007/s12272-017-0954-6, 28940173

[ref21] Liu L. Jiang Y. Steinle J. J. (2019). Glycyrrhizin protects the diabetic retina against permeability, neuronal, and vascular damage through anti-inflammatory mechanisms. J. Clin. Med. 8:957. doi: 10.3390/jcm8070957, 31269685 PMC6678129

[ref22] Liu Y. Wang Y. Yang Y. Quan Y. Guo M. (2024). Liquiritigenin induces cell cycle arrest and apoptosis in lung squamous cell carcinoma. Cell Biochem. Biophys. 82, 1397–1407. doi: 10.1007/s12013-024-01294-w 38775930

[ref23] Magwaza B. Amobonye A. Pillai S. (2024). Microbial β-glucosidases: recent advances and applications. Biochimie 22, 549–567. doi: 10.1016/j.biochi.2024.05.009 38734124

[ref24] Mariñas-Collado I. Rivas-López M. J. Rodríguez-Díaz J. M. Santos-Martín M. T. (2019). Optimal designs in enzymatic reactions with high-substrate inhibition. Chemom. Intell. Lab. Syst. 189, 102–109. doi: 10.1016/j.chemolab.2019.04.005

[ref25] Markina Y. V. Kirichenko T. V. Markin A. M. Yudina I. Y. Starodubova A. V. Sobenin I. A. . (2022). Atheroprotective effects of *Glycyrrhiza glabra* L. Molecules 27:4697. doi: 10.3390/molecules27154697, 35897875 PMC9332620

[ref26] Mittal A. Nagpal M. Vashistha V. K. (2023). Recent advances in the pharmacological activities of glycyrrhizin, Glycyrrhetinic acid, and their Analogs. Rev. Bras 33, 1154–1169. doi: 10.1007/s43450-023-00451-1

[ref27] Moon S. Ham S. Jeong J. Ku H. Kim H. Lee C. (2023). Temperature matters: bacterial response to temperature change. J. Microbiol. 61, 343–357. doi: 10.1007/s12275-023-00031-x 37010795

[ref28] Mou S. Zhou Z. Feng H. Zhang N. Lin Z. Aiyasiding X. . (2021). Liquiritin attenuates lipopolysaccharides-induced cardiomyocyte injury via an amp-activated protein kinase-dependent Signaling pathway. Front. Pharmacol. 12:648688. doi: 10.3389/fphar.2021.648688 34054527 PMC8162655

[ref29] Nascimento M. H. M. D. de Araújo D. R. (2022). Exploring the pharmacological potential of Glycyrrhizic acid: from therapeutic applications to trends in nanomedicine. Future Pharmacol. 2, 1–15. doi: 10.3390/futurepharmacol2010001

[ref30] Park C. Kim G. Cha G. (2021). Biotransformation of flavonoids by newly isolated and characterized *Lactobacillus pentosus* Ngi01 strain from kimchi. Microorganisms 9:1075. doi: 10.3390/microorganisms9051075, 34067804 PMC8157076

[ref31] Peterson M. E. Daniel R. M. Danson M. J. Eisenthal R. (2007). The dependence of enzyme activity on temperature: determination and validation of parameters. Biochem. J. 402, 331–337. doi: 10.1042/BJ20061143, 17092210 PMC1798444

[ref32] Rasool A. Dar T. A. (2025). Glycyrrhizin and its derivatives: an emerging secondary metabolite arsenal of Glycyrrhiza glabra. Med. Chem. Res. 34, 745–763. doi: 10.1007/s00044-025-03376-7

[ref33] Robinson P. K. (2015). Enzymes: principles and biotechnological applications. Essays Biochem. 59, 1–41. doi: 10.1042/bse0590001 26504249 PMC4692135

[ref34] Roncarati D. Vannini A. Scarlato V. (2024). Temperature sensing and virulence regulation in pathogenic bacteria. Trends Microbiol. 33, 66–79. doi: 10.1016/j.tim.2024.07.009 39164134

[ref35] Sahin A. Weilandt D. R. Hatzimanikatis V. (2023). Optimal enzyme utilization suggests that concentrations and thermodynamics determine binding mechanisms and enzyme saturations. Nat. Commun. 14:2618. doi: 10.1038/s41467-023-38159-4, 37147292 PMC10162984

[ref36] Sawadpongpan S. Jaratsittisin J. Hitakarun A. Roytrakul S. Wikan N. Smith D. R. (2023). Investigation of the activity of baicalein towards zika virus. BMC Complementary Med. Ther. 23:143. doi: 10.1186/s12906-023-03971-4 PMC10158012 37138273

[ref37] Segers M. E. Lebeer S. (2014). Towards a better understanding of *Lactobacillus rhamnosus* gg - host interactions. Microb. Cell Factories 13:S7. doi: 10.1186/1475-2859-13-S1-S7, 25186587 PMC4155824

[ref38] Selyutina O. Y. Polyakov N. E. (2019). Glycyrrhizic acid as a multifunctional drug carrier – from physicochemical properties to biomedical applications: a modern insight on the ancient drug. Int. J. Pharm. 55, 9271–9279. doi: 10.1016/j.ijpharm.2019.01.047 PMC7126914 30690130

[ref39] Semwal D. K. Kumar A. Semwal R. B. Dadhich N. K. Chauhan A. Kumar V. (2025). Glycyrrhizin (Glycyrrhizic acid)—pharmacological applications and associated molecular mechanisms. Drugs Drug Cand. 4:44. doi: 10.3390/ddc4040044

[ref40] Setlow B. Cabrera-Hernandez A. Cabrera-Martinez R. M. Setlow P. (2004). Identification of aryl-phospho-d-glucosidases in *Bacillus subtilis*. Arch. Microbiol. 181, 60–67. doi: 10.1007/s00203-003-0628-2, 14652714

[ref41] Stergiou O. S. Tegopoulos K. Kiousi D. E. Tsifintaris M. Papageorgiou A. C. Tassou C. C. . (2021). Whole-genome sequencing, phylogenetic and genomic analysis of Lactiplantibacillus pentosus L33, a potential probiotic strain isolated from fermented sausages. Front. Microbiol. 12:746659. doi: 10.3389/fmicb.2021.746659 34764945 PMC8576124

[ref42] Stoffel F. Papp M. Gil-Garcia M. Küffner A. M. Benítez-Mateos A. I. Jacquat R. P. B. . (2025). Enhancement of enzymatic activity by biomolecular condensates through pH buffering. Nat. Commun. 16:6368. doi: 10.1038/s41467-025-61013-8 40640131 PMC12246476

[ref43] Sun X. Zeng H. Wang Q. Yu Q. Wu J. Feng Y. . (2018). Glycyrrhizin ameliorates inflammatory pain by inhibiting microglial activation-mediated inflammatory response via blockage of the Hmgb1-Tlr4-Nf-kB pathway. Exp. Cell Res. 369, 112–119. doi: 10.1016/j.yexcr.2018.05.012, 29763588

[ref44] Vanella R. Küng C. Schoepfer A. A. Doffini V. Ren J. Nash M. A. (2024). Understanding activity-stability tradeoffs in biocatalysts by enzyme proximity sequencing. Nat. Commun. 15:1807. doi: 10.1038/s41467-024-45630-3 38418512 PMC10902396

[ref45] Wahab S. Annadurai S. Abullais S. S. Das G. Ahmad W. Ahmad M. F. . (2021). *Glycyrrhiza glabra* (Licorice): A comprehensive review on its phytochemistry, biological activities, clinical evidence and toxicology. Plants 10:2751. doi: 10.3390/plants10122751, 34961221 PMC8703329

[ref46] Woelfel S. Silva M. S. Stecher B. (2024). Intestinal colonization resistance in the context of environmental, host, and microbial determinants. Cell Host Microbe 32, 820–836. doi: 10.1016/j.chom.2024.05.002, 38870899

[ref47] Wu L. Ma T. Zang C. Xu Z. Sun W. Luo H. . (2024). Glycyrrhiza, a commonly used medicinal herb: review of species classification, pharmacology, active ingredient biosynthesis, and synthetic biology. J. Adv. Res. 75, 249–270. doi: 10.1016/j.jare.2024.11.019 39551128 PMC12536610

[ref48] Wu Y. Wang Z. Du Q. Zhu Z. Chen T. Xue Y. . (2022). Pharmacological effects and underlying mechanisms of Licorice-derived flavonoids. Evid. Based Complement. Alternat. Med. 20, 221–225. doi: 10.1155/2022/9523071 PMC8786487 35082907

[ref49] Ye K. Li P. Gu Q. (2020). Complete genome sequence analysis of a strain *Lactobacillus pentosus* Zfm94 and its probiotic characteristics. Genomics 112, 3142–3149. doi: 10.1016/j.ygeno.2020.05.015, 32450257

[ref50] Yuan T. Wang J. Chen L. Shan J. Di L. (2019). Glycyrrhizic acid improving the liver protective effect by restoring the composition of Lactobacillus. J. Funct. Foods 52, 219–227. doi: 10.1016/j.jff.2018.11.001

[ref51] Zapata-Torres G. Fierro A. Miranda-Rojas S. Guajardo C. Saez-Briones P. Salgado J. C. . (2012). Influence of protonation on substrate and inhibitor interactions at the active site of human monoamine oxidase-A. J. Chem. Inf. Model. 52, 1213–1221. doi: 10.1021/ci300081w, 22540832

[ref52] Zhou M. Cai Q. Zhang C. Ouyang P. Yu L. Xu Y. (2022). Antibiotic resistance bacteria and antibiotic resistance genes survived from the extremely acidity posing a risk on intestinal bacteria in an in vitro digestion model by horizontal gene transfer. Ecotoxicol. Environ. Saf. 247:114247. doi: 10.1016/j.ecoenv.2022.114247 36332408

