# Supplementary material for: Complete genome sequencing of Lactobacillus pentosus HP-B1718 and identification of the key enzyme for liquiritin biotransformation
Source: Front Microbiol. 2026 May 20;17:1840719. doi: 10.3389/fmicb.2026.1840719 (PMC13231499; doi:10.3389/fmicb.2026.1840719)
Supplement: Supplementary file 1 [file Supplementary_File_1.DOCX]

Supplementary Material

# Supplementary Figures and Tables

## Supplementary Tables

**Supplementary Table 1.** Statistics of coding gene annotation results

| Item | Count | Percentage |
| --- | --- | --- |
| All | 3,081 | 100.00% |
| Annotation | 3,080 | 99.97% |
| KEGG | 2,336 | 75.82% |
| Pathway | 1,160 | 37.65% |
| Nr | 3,080 | 99.97% |
| Uniprot | 3,073 | 99.74% |
| GO | 2,348 | 76.21% |
| COG | 2,540 | 82.44% |
| Pfam | 2,532 | 82.18% |
| Refseq | 3,051 | 99.03% |
| Tigerfam | 1,694 | 54.98% |

**Supplementary Table 2**. Thermal tolerance of *Lactobacillus pentosus* HP-B1718

| Temperature (℃) | Viable count (CFU/mL) | | |
| --- | --- | --- | --- |
|  | 1 min | 2 min | 3 min |
| 37℃ | 2.19×10^9^ | 2.11×10^9^ | 2.07×10^9^ |
| 45℃ | 2.03×10^9^ | 2.37×10^9^ | 1.72×10^9^ |
| 50℃ | 1.99×10^9^ | 1.18×10^9^ | 4.80×10^8^ |

**Supplementary Table 3.** Study on Acid Tolerance of *Lactobacillus pentosus* HP-B1718

| pH | Time(min) | Viable count/(CFU/mL) | Survival rate (%) |
| --- | --- | --- | --- |
| 2.30 | 0 | 1.03×10^9^ | - |
|  | 150 | 3.41×10^9^ | 331.06% |
| 3.24 | 0 | 1.00×10^9^ | - |
|  | 150 | 3.31×10^9^ | 331.00% |
| 4.31 | 0 | 5.3×10^8^ | - |
|  | 150 | 1.98×10^9^ | 373.58% |

**Supplementary Table 4.** Study on Bile Salt Tolerance of *Lactobacillus pentosus* HP-B1718

| Cholesterol content (%) | Time (min) | Viable count /(CFU/mL) | Survival rate (%) |
| --- | --- | --- | --- |
| 0.03 | 0 | 2.46×10^9^ | 184.14% |
|  | 150 | 4.53×10^9^ |  |
| 0.1 | 0 | 2.40×10^9^ | 156.25% |
|  | 150 | 3.75×10^9^ |  |
| 0.2 | 0 | 2.25×10^9^ | 180.88% |
|  | 150 | 4.07×10^9^ |  |
| 0.3 | 0 | 1.97×10^9^ | 151.26% |
|  | 150 | 2.98×10^9^ |  |

**Supplementary Table 5.** Evaluation of Antibacterial Effect of *Lactobacillus pentosus* HP-B1718 Fermentation Broth

| **Pathogen Name** | **Sample Type** | **Incubation Time** | **Inhibition Zone Diameter** |
| --- | --- | --- | --- |
| *Salmonella typhi*  HP-B1155 | Fermentation Broth (F) | 20 | 21.68 |
|  | Fermentation Supernatant (S) | 20 | 20.58 |
| *Escherichia coli*  HP-B1156 | Fermentation Broth (F) | 20 | 21.52 |
|  | Fermentation Supernatant (S) | 20 | 20.98 |
| *Bacillus cereus*  HP-B1157 | Fermentation Broth (F) | 20 | 21.60 |
|  | Fermentation Supernatant (S) | 20 | 20.46 |
| *Klebsiella pneumoniae*  HP-B1158 | Fermentation Broth (F) | 20 | 19.00 |
|  | Fermentation Supernatant (S) | 20 | 19.34 |
| *Acinetobacter baumannii*  HP-B1160 | Fermentation Broth (F) | 20 | 20.60 |
|  | Fermentation Supernatant (S) | 20 | 19.44 |
| *Escherichia coli*  HP-B1161 | Fermentation Broth (F) | 20 | 18.82 |
|  | Fermentation Supernatant (S) | 20 | 18.42 |
| *Proteus mirabilis*  HP-B1162 | Fermentation Broth (F) | 20 | 20.80 |
|  | Fermentation Supernatant (S) | 20 | 20.54 |
| Drug-resistant Escherichia coli  HP-B1163 | Fermentation Broth (F) | 20 | 17.86 |
|  | Fermentation Supernatant (S) | 20 | 18.64 |
| Drug-resistant Acinetobacter baumannii  HP-B1164 | Fermentation Broth (F) | 20 | 19.02 |
|  | Fermentation Supernatant (S) | 20 | 19.34 |
| Drug-resistant Acinetobacter baumannii  HP-B1165 | Fermentation Broth (F) | 20 | 16.10 |
|  | Fermentation Supernatant (S) | 20 | 16.78 |
| Drug-resistant Pseudomonas aeruginosa  HP-B1166 | Fermentation Broth (F) | 20 | 19.16 |
|  | Fermentation Supernatant (S) | 20 | 19.24 |
| Drug-resistant Staphylococcus aureus  HP-B1167 | Fermentation Broth (F) | 20 | 19.66 |
|  | Fermentation Supernatant (S) | 20 | 20.04 |
| Shigella flexneri  HP-B1168 | Fermentation Broth (F) | 20 | 19.94 |
|  | Fermentation Supernatant (S) | 20 | 20.48 |
| DH5a  HP-B1169 | Fermentation Broth (F) | 20 | 19.02 |
|  | Fermentation Supernatant (S) | 20 | 1.896 |
| BL21  HP-B1170 | Fermentation Broth (F) | 20 | 19.30 |
|  | Fermentation Supernatant (S) | 20 | 20.46 |
| Shigella sonnei  HP-B1171 | Fermentation Broth (F) | 20 | 18.02 |
|  | Fermentation Supernatant (S) | 20 | 18.34 |

**Supplementary Table 6.** HPLC external standard method. the peak areas and corresponding contents of liquiritin at each concentration gradient

| **Liquiritin Concentration Gradient** | **Liquiritin Peak Area (Mean ± SD)** | **Liquiritin Content (mg/L)** |
| --- | --- | --- |
| 16-fold group | 577.64 ± 38.23 | 62.50 |
| 8-fold group | 1255.72 ± 50.70 | 125.00 |
| 6-fold group | 1564.24 ± 51.25 | 166.67 |
| 4-fold group | 2458.29 ± 24.93 | 250.00 |
| 2-fold group | 4862.89 ± 36.89 | 500.00 |
| 1.5-fold group | 6563.26 ± 19.63 | 666.67 |

**Supplementary Table 7.** Analysis of variance (ANOVA) for the regression model

| Soruce of variation | Type III sum of squares | Degree of freedom | Mean square | F | P |
| --- | --- | --- | --- | --- | --- |
| Modified model | 4772625.344350 | 23 | 18.053259 | 18.053259 | <0.05 |
| Intercept | 18268896.811250 | 1 | 1589.419079 | 1589.419079 | <0.05 |
| b | 1630950.169000 | 5 | 28.378980 | 28.378980 | <0.05 |
| a | 2795396.512550 | 3 | 81.067777 | 81.067777 | <0.05 |
| a*b | 346278.662800 | 15 | 2.008448 | 2.008448 | 0.035 |
| Error | 551715.440400 | 48 |  |  |  |
| Aggregate | 23593237.596000 | 72 |  |  |  |

## Supplementary Figures

**
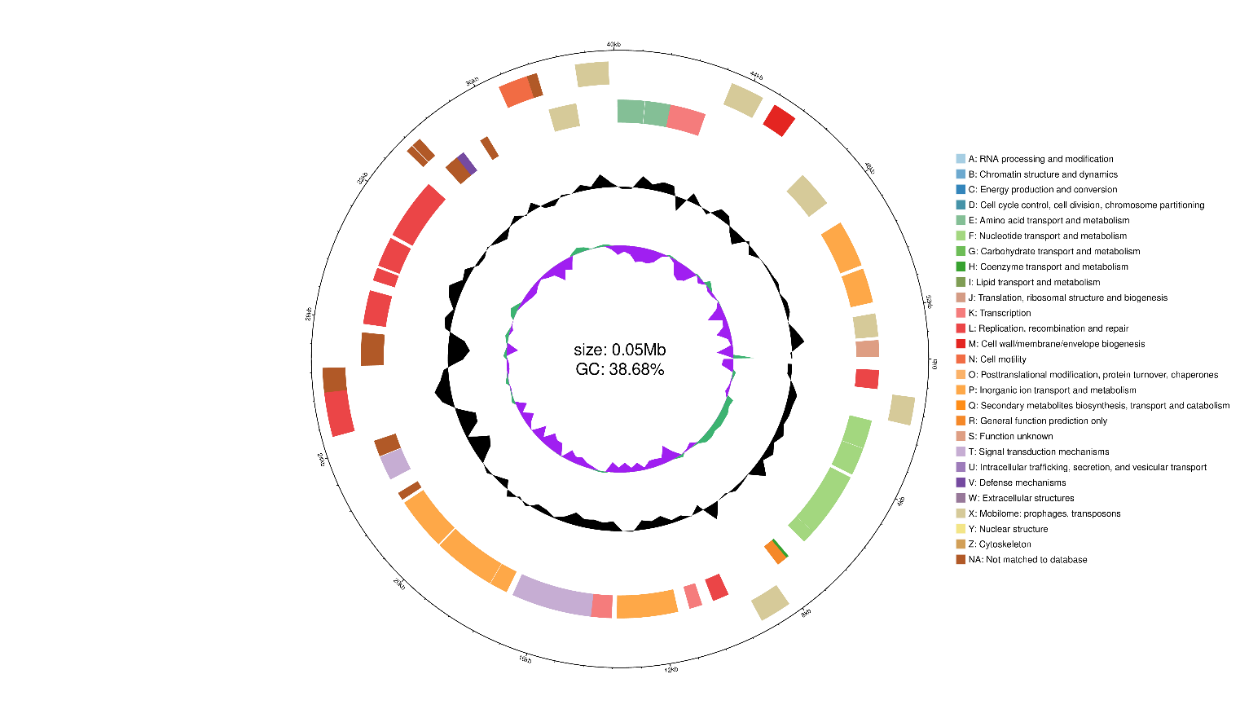
**

**Supplementary Figure 1.** Circular plasmid map of *Lactobacillus pentosus* HP-B1718. From outside to inside: the first circle, genome coordinates. the second circle, genes on the positive strand of the genome sequence, with different colors representing different COG functional classifications. the third circle, genes on the negative strand of the genome sequence, with different colors representing different COG functional classifications. the fourth circle, rRNA and tRNA in the genome sequence, rRNA in blue and tRNA in red. the fifth circle, G+C content curve with a 2000 bp sliding window. the sixth circle, G+C skew curve with a 2000 bp sliding window.


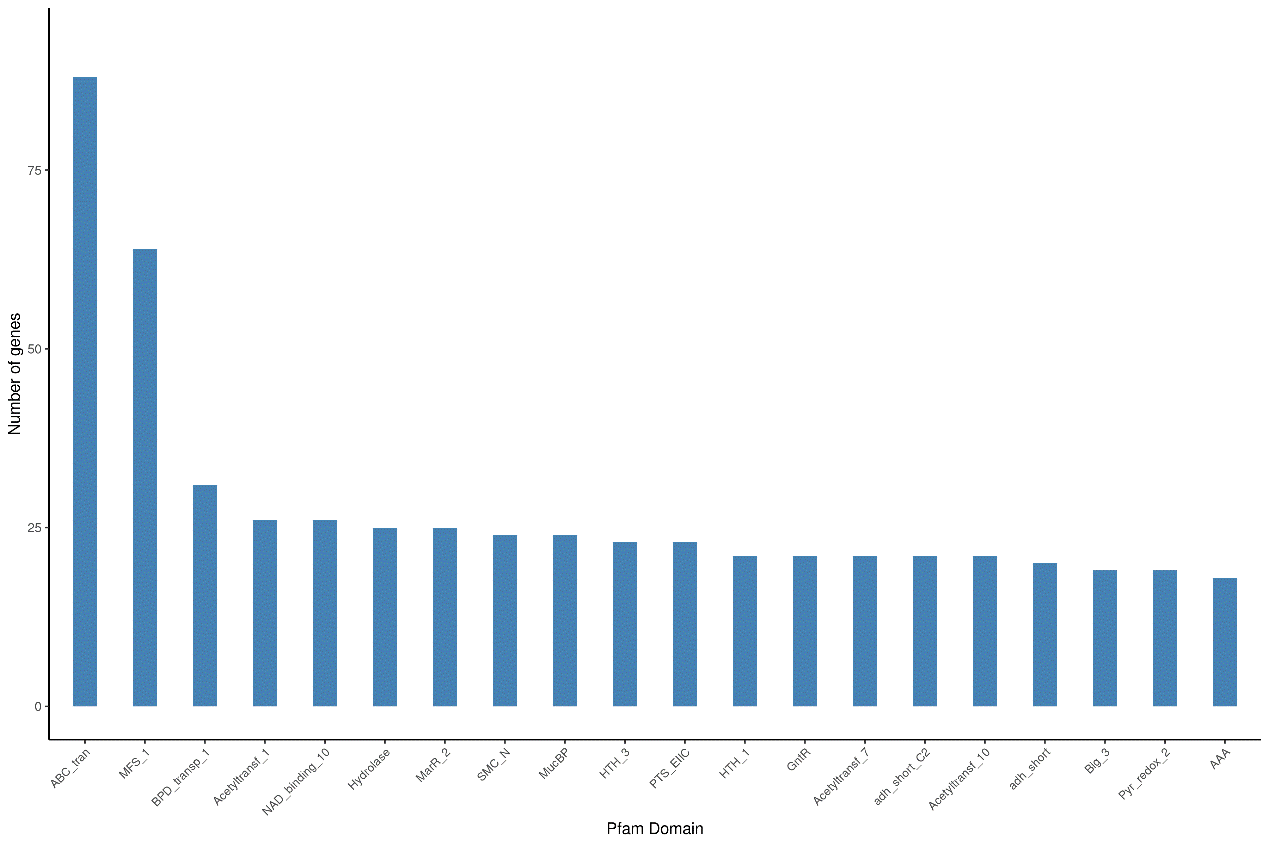


**Supplementary Figure 2.** The abscissa is the name of the protein family of *Lactobacillus pentosus* HP-B1718, and the ordinate is the number of genes aligned to the protein family.


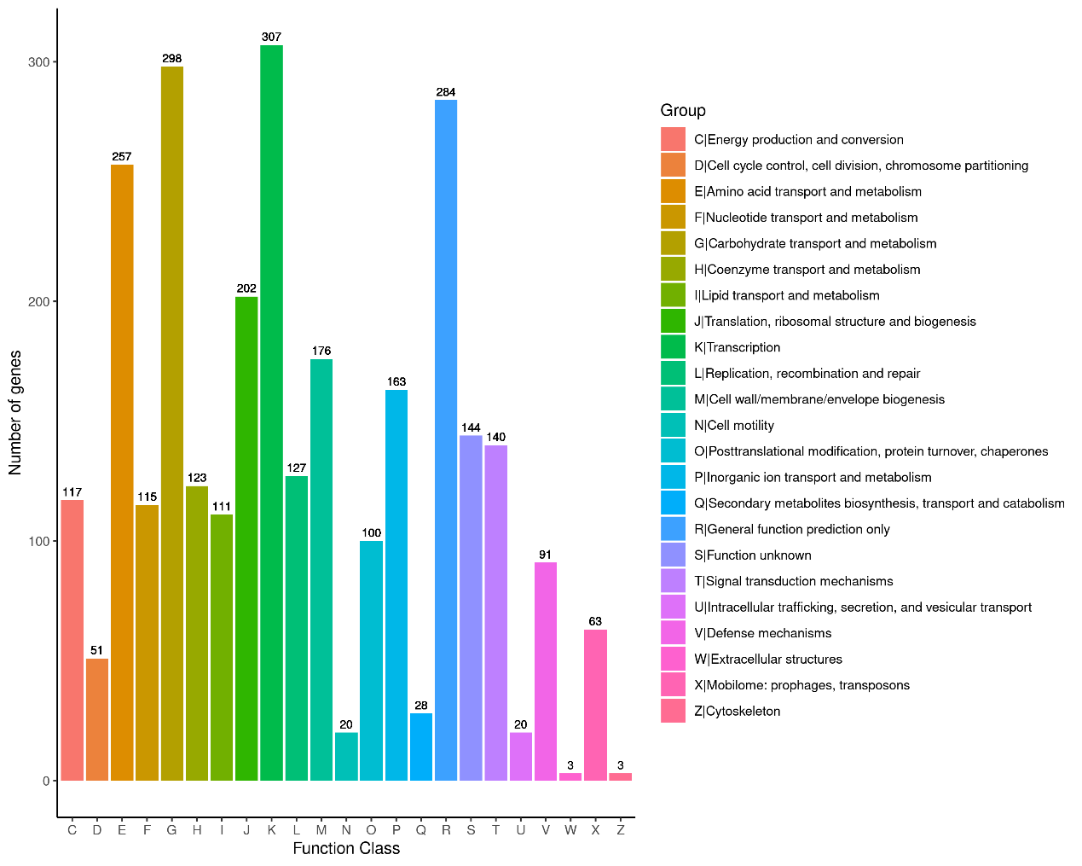


**Supplementary Figure 3.** The abscissa is the content of each COG classification, and the ordinate is the number of genes. The number of genes reflects the metabolic or physiological tendency in the corresponding period and environment, and scientific explanations can be made by combining the distribution of the research object in each functional category.


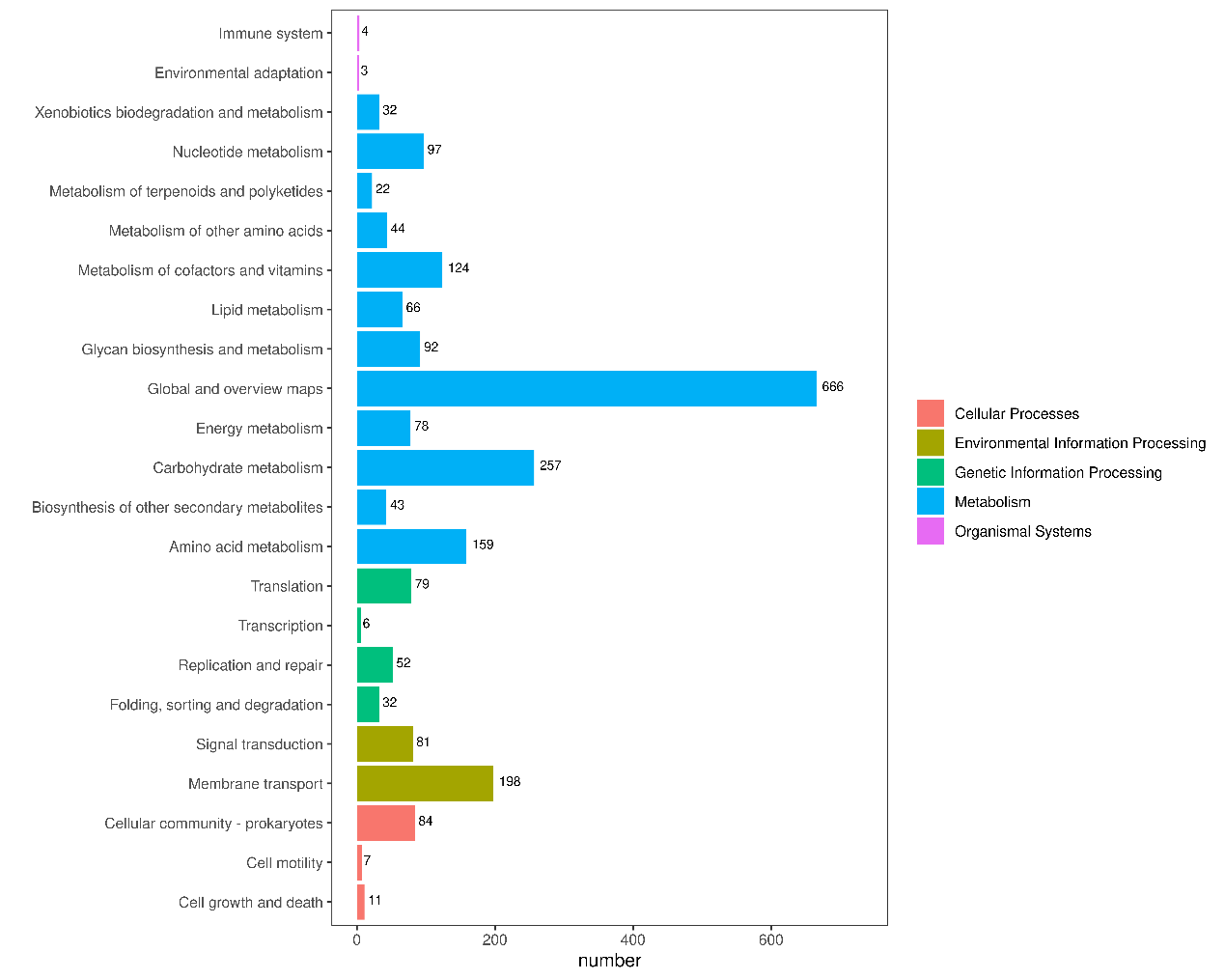


**Supplementary Figure 4.** The abscissa is the number of genes annotated under Pathway classification, the ordinate is Pathway classification, with different colors representing different major classifications.


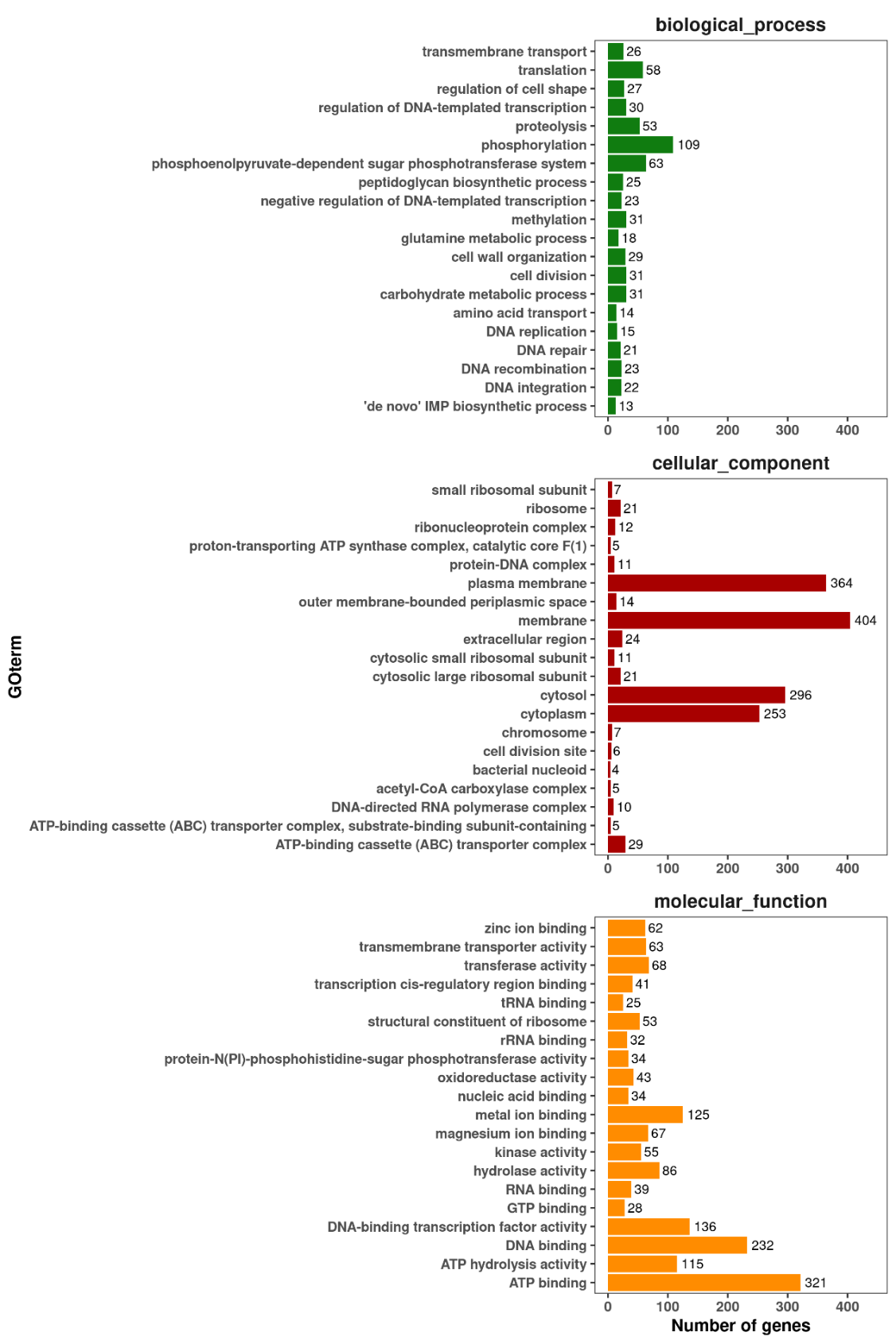


**Supplementary Figure 5.** The abscissa is the content of each GO classification, and the ordinate is the number of genes.


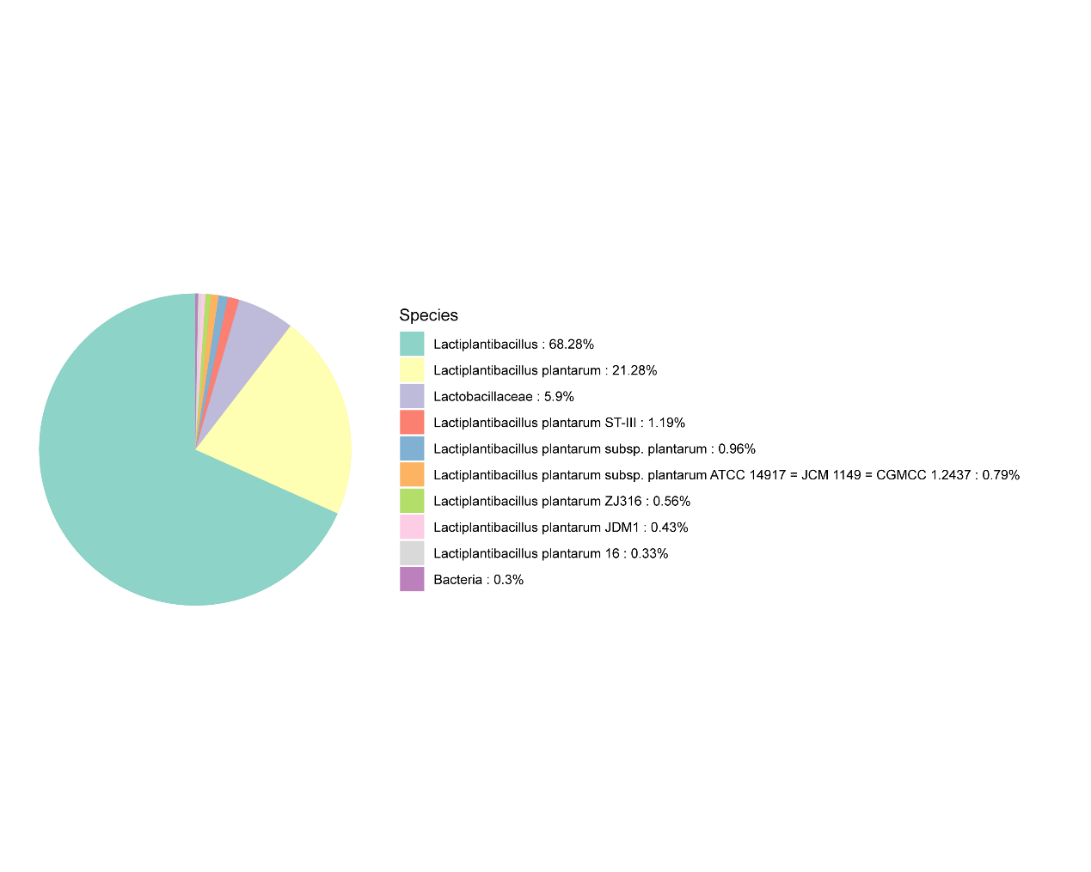


**Supplementary Figure 6.** NR species distribution map, with different colors representing different species in the figure.


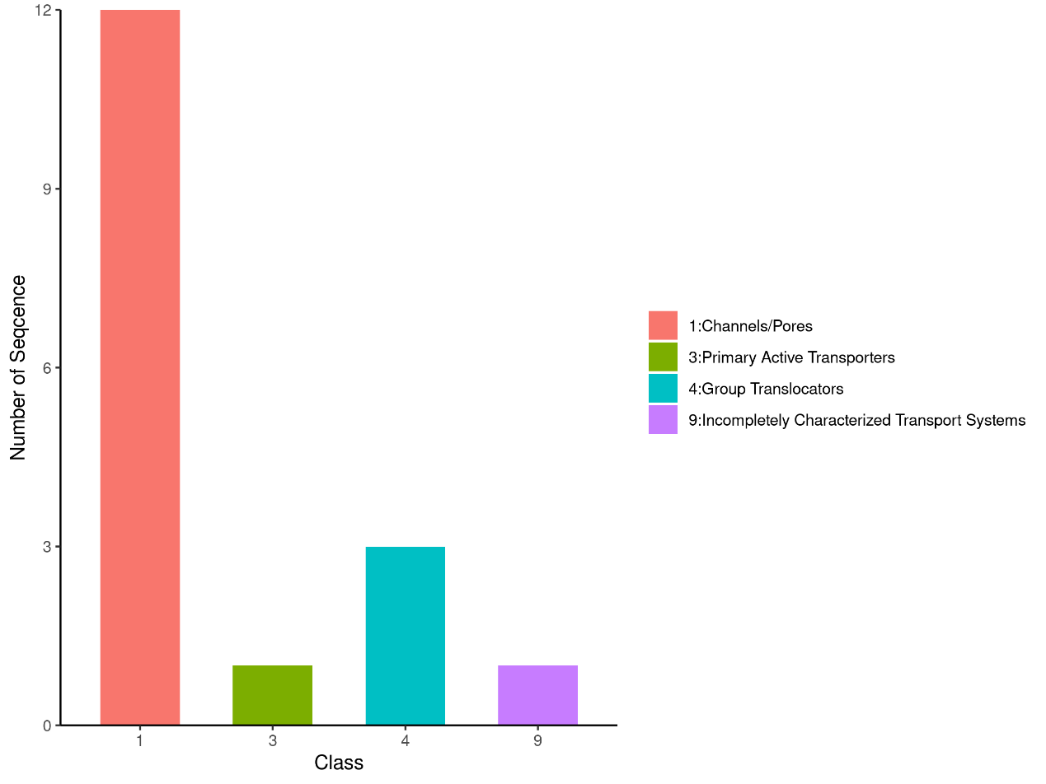


**Supplementary Figure 7.** Genome annotation of *Lactobacillus pentosus* HP-B1718 classified by TCDB (Transporter Classification Database).


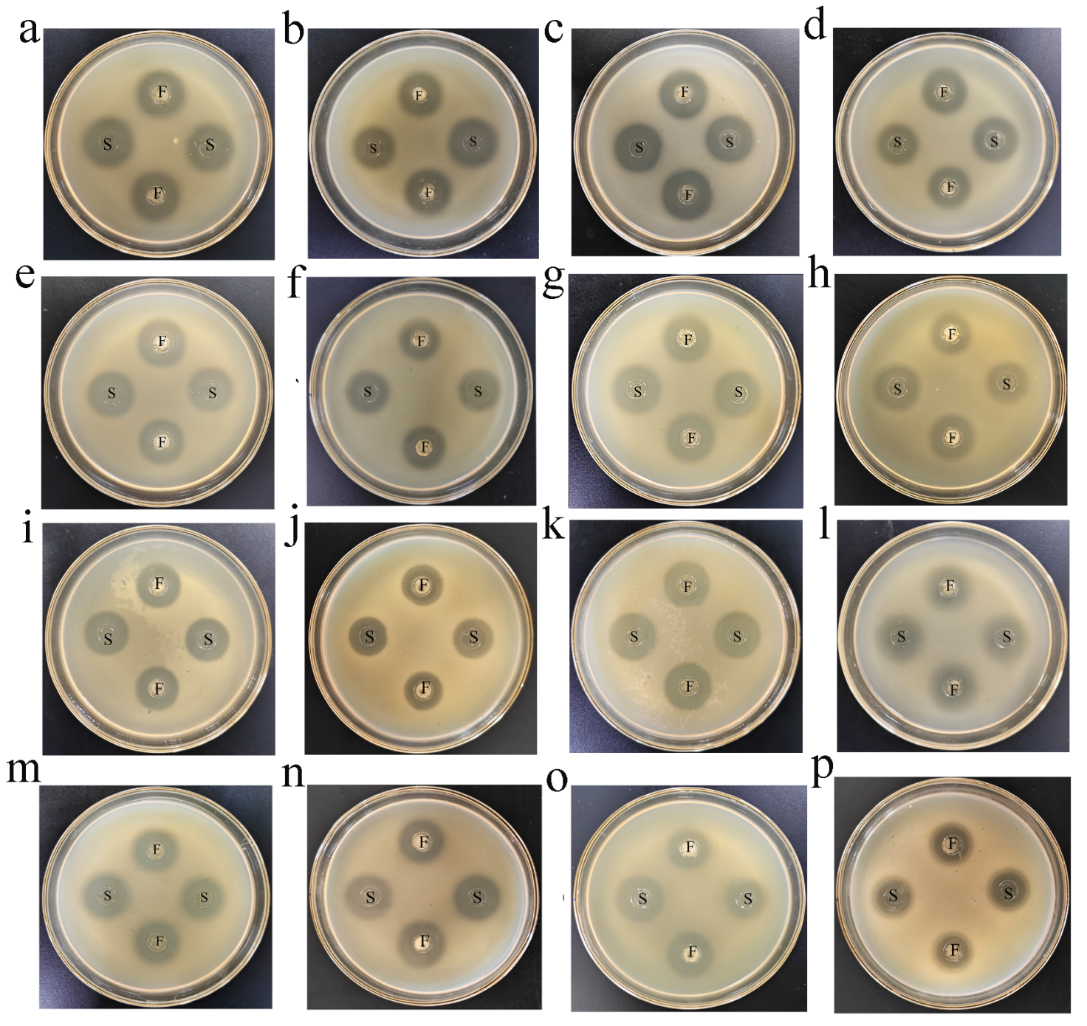


**Supplementary Figure 8.** Antibacterial effects of *Lactobacillus pentosus* HP‑B1718 fermentation broth against various human pathogenic bacteria detected by the Oxford cup method. A-P represent the antibacterial effects on *Salmonella typhi* HP‑B1155, *Escherichia coli* HP‑B1156, *Bacillus cereus* HP‑B1157, *Klebsiella pneumoniae* HP‑B1158, *Acinetobacter baumannii* HP‑B1160, *Escherichia coli* HP‑B1161, *Proteus mirabilis* HP‑B1162, drug‑resistant *Escherichia coli* HP‑B1163, drug‑resistant *Acinetobacter baumannii* HP‑B1164, drug‑resistant *Acinetobacter baumannii* HP‑B1165, drug‑resistant *Pseudomonas aeruginosa* HP‑B1166, drug‑resistant *Staphylococcus aureus* HP‑B1167, *Shigella flexneri* HP‑B1168, DH5α HP‑B1169, BL21 HP‑B1170, and *Shigella sonnei* HP‑B1171, respectively. F: uncentrifuged fermentation broth; S: centrifuged supernatant.

**Supplementary Figure 9.** For HPLC quantification, the external standard method was used. A series of standard solutions of liquiritin with varying concentrations were prepared, and their peak areas were determined. A standard curve was established by linear regression of peak area against the mass concentration of liquiritin.

**
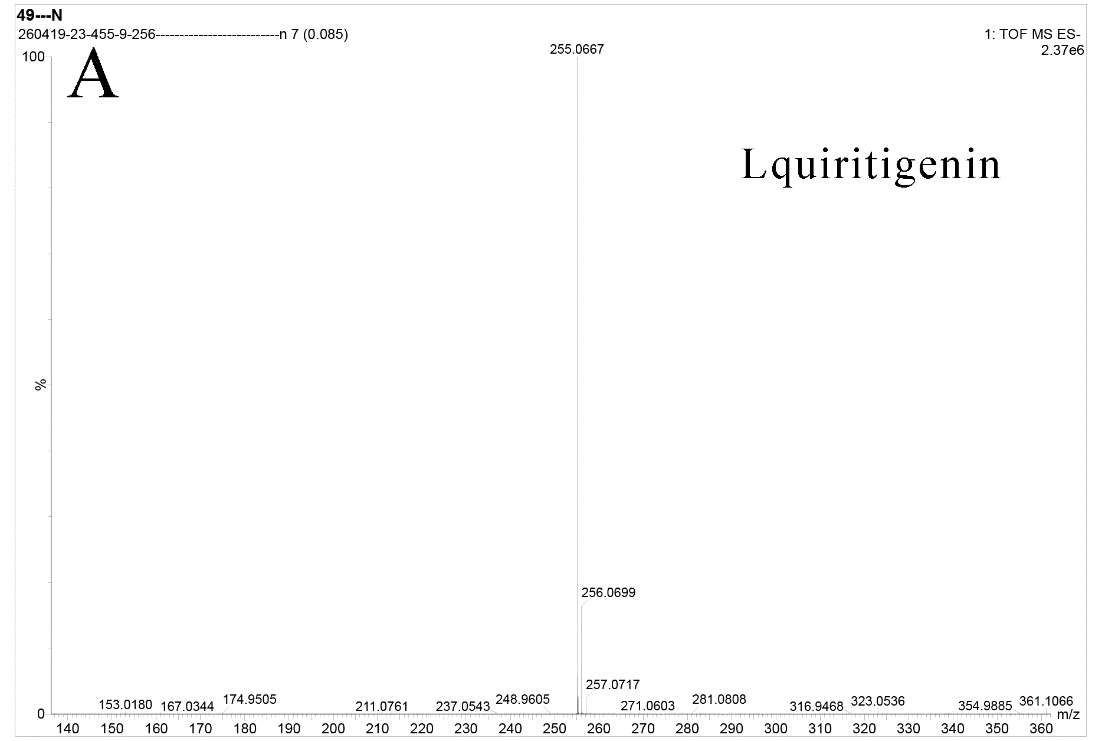
**

**Supplementary Figure 10A.** The mass spectrum of the liquiritigenin standard exhibited a quasi-molecular ion peak at m/z 255.0667 [M−H]⁻, which was highly consistent with the calculated value (m/z 255.0657) for the theoretical formula C₁₅H₁₁O₄⁻, with a mass error of only 3.9 ppm. Thus, the molecular formula of liquiritigenin was confirmed to be C₁₅H₁₂O₄.

**
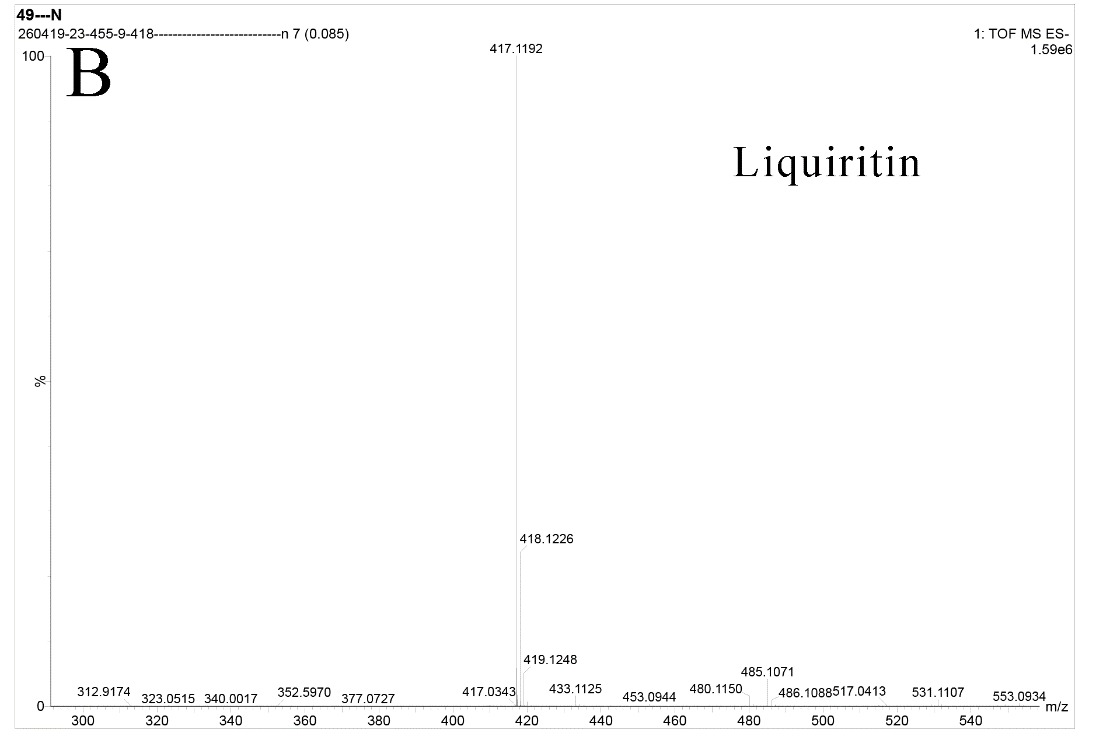
**

**Supplementary Figure 10B.** For the liquiritin standard, the quasi-molecular ion peak appeared at *m/z* 417.1192 [M-H]⁻, which matched the calculated value for C₂₁H₂₁O₉⁻ (*m/z* 417.1186) with a mass error of 1.4 ppm, verifying its molecular formula as C₂₁H₂₂O₉.

**
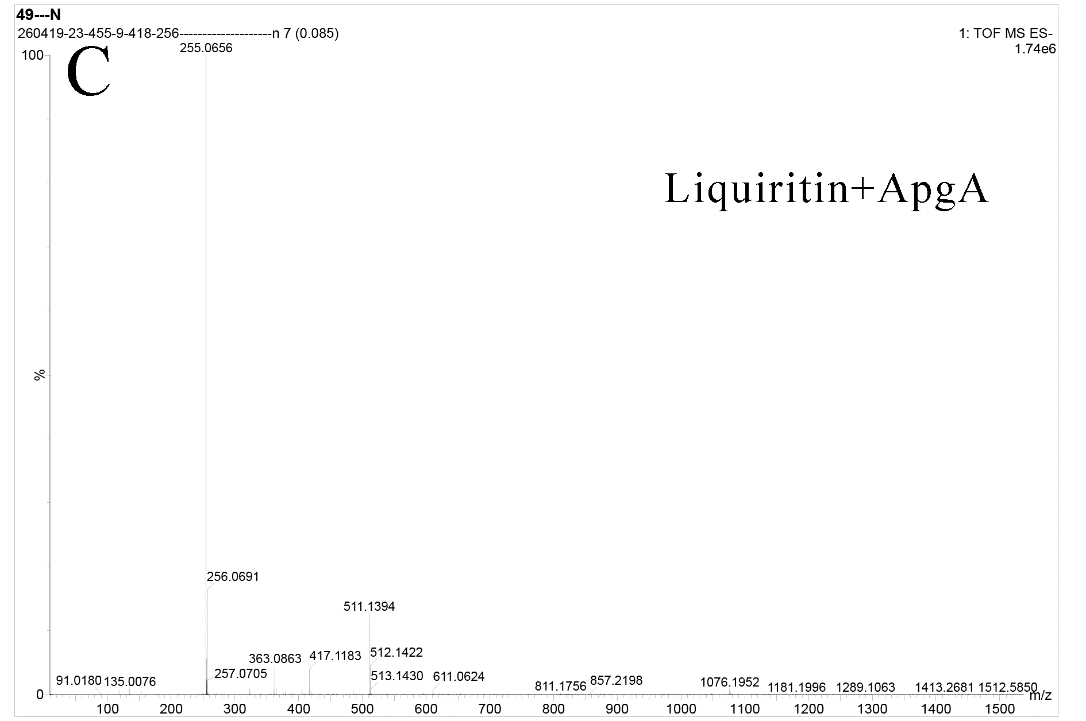
**

**Supplementary Figure 10C.** Both the substrate and product were simultaneously detected in the reaction mixture of liquiritin and ApgA (Figure 9C). The quasi-molecular ion peak corresponding to liquiritin was observed at *m/z* 417.1183 [M-H]⁻, and that of the product liquiritigenin at *m/z* 255.0656 [M-H]⁻. These two ions coexisted in the sample.


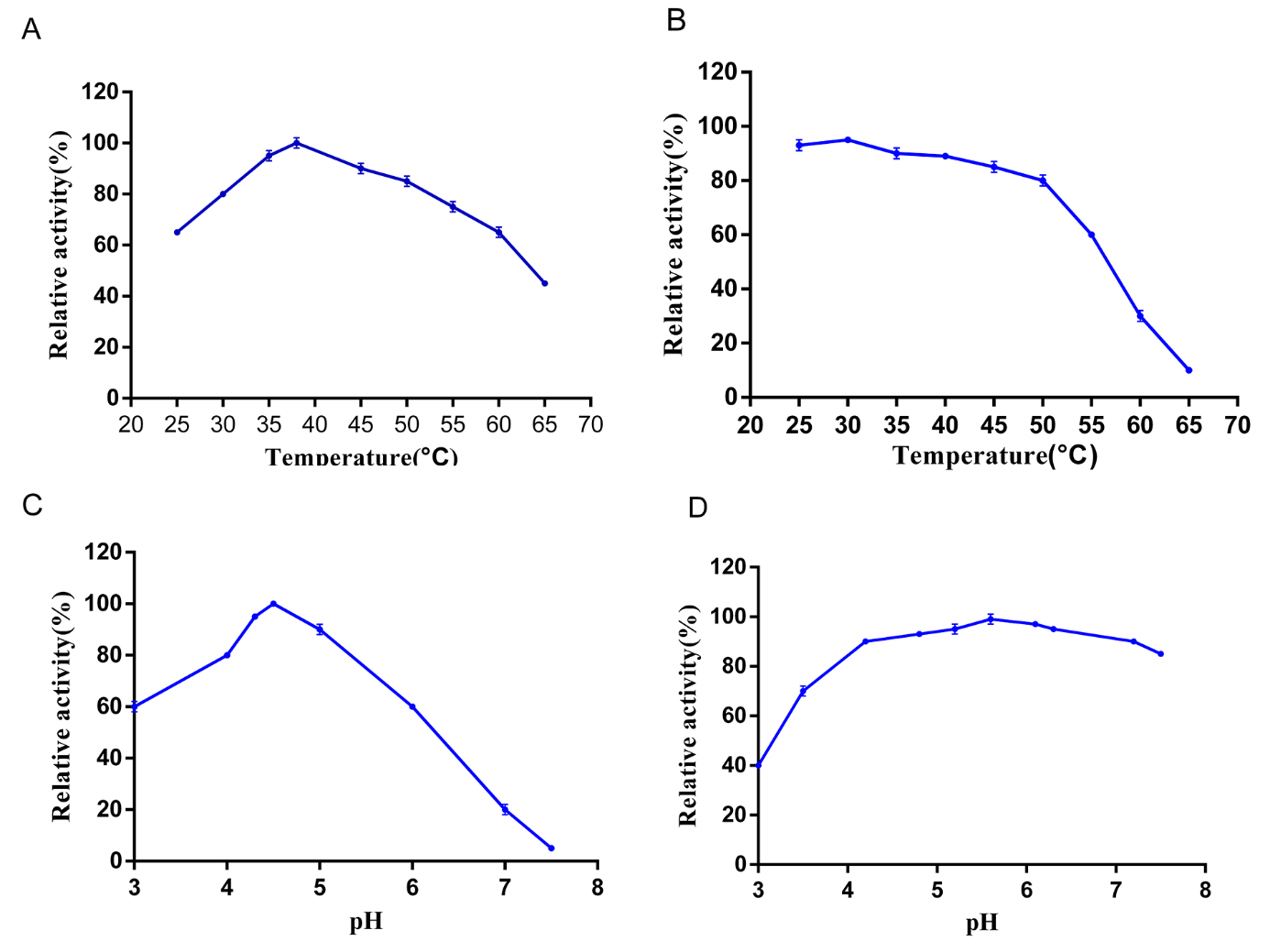


**Supplementary Figure 11.** Characterization of the enzymatic properties of recombinant ApgAenzyme. A, Optimal temperature. The enzyme activity at 38 °C was taken as 100%. B, Thermal stability. The residual enzyme activity was determined after incubating the enzyme at pH 4.5 and different temperatures for 12 h. the enzyme activity before incubation (0 h) was taken as 100%. C, Optimal pH. The enzyme activity at pH 4.5 was taken as 100%. D, pH stability. The residual enzyme activity was determined after dialyzing the enzyme at 4 °C and different pH conditions for 12 h. the enzyme activity before dialysis (0 h) was taken as 100%.
